# Rewiring Neuronal Glycerolipid Metabolism Determines the Extent of Axon Regeneration

**DOI:** 10.1016/j.neuron.2019.10.009

**Published:** 2020-01-22

**Authors:** Chao Yang, Xu Wang, Jianying Wang, Xuejie Wang, Weitao Chen, Na Lu, Symeon Siniossoglou, Zhongping Yao, Kai Liu

**Affiliations:** 1 Division of Life Science, State Key Laboratory of Molecular Neuroscience, The Hong Kong University of Science and Technology, Hong Kong, China; 2 Shenzhen Key Laboratory for Neuronal Structural Biology, Biomedical Research Institute, Shenzhen Peking University–The Hong Kong University of Science and Technology Medical Center, Shenzhen 518036, China; 3 State Key Laboratory of Chirosciences, Food Safety and Technology Research Centre and Department of Applied Biology and Chemical Technology, The Hong Kong Polytechnic University, Hong Kong, China; 4 Cambridge Institute for Medical Research, University of Cambridge, Cambridge CB2 0XY, UK; 5 Center of Systems Biology and Human Health, School of Science and Institute for Advanced Study, The Hong Kong University of Science and Technology, Hong Kong, China

**Keywords:** axon regeneration, retinal ganglion cell, glycerolipid, triglyceride, phospholipid, Lipin1, DGAT1, DGAT2

## Abstract

How adult neurons coordinate lipid metabolism to regenerate axons remains elusive. We found that depleting neuronal lipin1, a key enzyme controlling the balanced synthesis of glycerolipids through the glycerol phosphate pathway, enhanced axon regeneration after optic nerve injury. Axotomy elevated lipin1 in retinal ganglion cells, which contributed to regeneration failure in the CNS by favorably producing triglyceride (TG) storage lipids rather than phospholipid (PL) membrane lipids in neurons. Regrowth induced by lipin1 depletion required TG hydrolysis and PL synthesis. Decreasing TG synthesis by deleting neuronal diglyceride acyltransferases (DGATs) and enhancing PL synthesis through the Kennedy pathway promoted axon regeneration. In addition, peripheral neurons adopted this mechanism for their spontaneous axon regeneration. Our study reveals a critical role of lipin1 and DGATs as intrinsic regulators of glycerolipid metabolism in neurons and indicates that directing neuronal lipid synthesis away from TG synthesis and toward PL synthesis may promote axon regeneration.

## Highlights

• Injury-elevated Lipin1 and DGAT in retinal ganglion cells suppress regeneration
• Neuronal lipin1 and DGATs increase triglyceride and decrease phospholipids
• Redirecting triacylglyceride to phospholipid synthesis promotes axon regeneration

## Graphical Abstract

**Figure:**
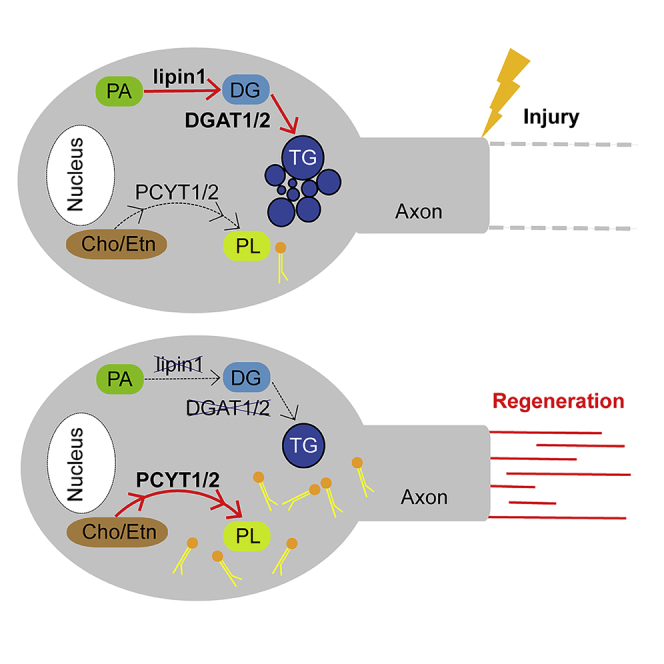

## Teaser

Axotomy alters several glycerolipid biosynthetic enzymes to regulate axon regeneration in both CNS and PNS neurons. Depleting neuronal lipin1 and DGAT to redirect lipid synthesis away from triglycerides and toward phospholipids promotes axon regeneration.

## Introduction

Axon regeneration through modulating neuronal intrinsic mechanisms is a very promising strategy to develop potential therapies for neural repair after CNS injury (Fawcett and Verhaagen, 2018, He and Jin, 2016, Liu et al., 2011, Mahar and Cavalli, 2018). Understanding the basic biological processes within neurons that actively retard or enhance axon regrowth is becoming increasingly important (Curcio and Bradke, 2018, Goldberg, 2003). Injured neurons require a large supply of lipids for membrane formation as they grow long axons during regeneration (Bradke et al., 2012, Pfenninger, 2009, Vance et al., 2000). Many classes of lipids exist in neurons with various functions, and not necessarily all lipids are crucial for axon growth. Thus, axon regrowth requires coordinated changes in lipid homeostasis in injured neurons. The metabolism of lipids such as fatty acids and cholesterol has been actively studied in the brain (Bazinet and Layé, 2014, Pfrieger and Ungerer, 2011). Recent studies in *Drosophila* larvae sensory neurons indicate that neuronal lipid biosynthesis regulates dendritic complexity (Meltzer et al., 2017, Ziegler et al., 2017). However, relatively little is known about how lipid metabolism is intrinsically regulated in neurons to control axon elongation and regeneration.

Glycerolipids are abundant cellular lipids, including triglycerides (TGs) for energy storage and phospholipids (PLs) for membrane structure. Although TG molecules help organisms survive starvation, they are not regarded as a major direct source of energy for the brain (Schönfeld and Reiser, 2013). However, recent evidence suggests that neuronal TG lipases are very active and that TGs undergo constant turnover in adult neurons (Inloes et al., 2014). TG lipase hydrolyzes a TG to one fatty acid and one diglyceride (DG). DGs are also a precursor of TGs and PLs. Because PLs and TGs share common precursors, neurons likely utilize this strategy to direct the flow of lipids toward membrane production or energy storage depending on needs.

The glycerol phosphate pathway (glycerol 3-phosphate pathway) is an important mechanism for controlling the glycerolipid levels in cells by regulating a series of enzymatic reactions. Lipin1 protein, a phosphatidic acid phosphatase (PAP) enzyme, plays a central role in the penultimate step of the glycerol phosphate pathway and catalyzes the conversion of phosphatidic acid (PA) to DG (Han et al., 2006, Han et al., 2007). In addition, lipin1 can also regulate gene expression independent of its catalytic function by relocating to the nucleus and acting as a coregulator with transcription factors (Finck et al., 2006). Mutation of lipin1 causes lipodystrophy with almost complete loss of fat (Harris and Finck, 2011, Reue and Zhang, 2008). In the glycerol phosphate pathway, the final and only committed step is to form a TG by covalently joining a fatty acyl-CoA and a DG molecule. This reaction is catalyzed by two acyl-CoA:diacylglycerol acyltransferase (DGAT) enzymes, DGAT1 and DGAT2, both of which have been implicated in modulating TG homeostasis (Yen et al., 2008). The glycerol phosphate pathway is well characterized in tissues specialized for energy storage or lipid turnover, such as adipose tissue and liver. The function of this metabolic pathway in neuronal response to injury and morphological change, especially in regard to axon growth, has not been explored.

Neurons acquire lipid supplies either through uptake from the external environment or *de novo* biosynthesis. Regardless of where they are from, lipid building blocks must undergo metabolic processes before they can be utilized by neurons for various functions. We hypothesized that coordinated lipid metabolism plays a role in axon regeneration. Here, we report that neuronal lipin1 depletion promoted axon regeneration by regulating glycerolipid metabolism. Axotomy elevated lipin1 in retinal ganglion cells (RGCs), and this upregulation contributed to regeneration failure. Lipin1 depletion promoted axon regrowth by regulating TG hydrolysis and PL synthesis. Directly suppressing TG biosynthesis also promoted axon regeneration and reprogrammed glycerolipid metabolism in the same direction as lipin1 depletion. In contrast to RGCs, peripheral neurons downregulated DGAT1 upon axotomy, and TG hydrolysis was required for axon regeneration after sciatic nerve injury. Thus, we propose that TGs may provide lipid precursors to generate PLs for membrane biosynthesis during axon regeneration and that the glycerol phosphate pathway is a potential target for neural repair.

## Results

### Lipin1 Is an Intrinsic Suppressor of Axon Regeneration

**Figure 1:**
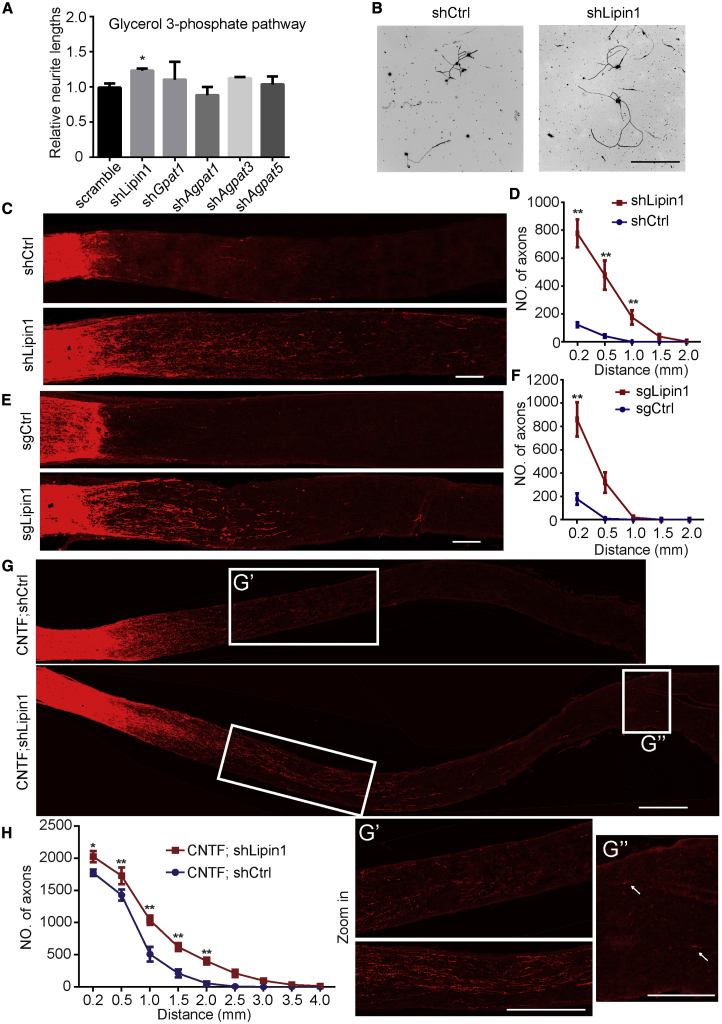
Lipin1 Depletion Promotes Axon Regeneration (A) Quantification of the axon elongation by *in vitro* screening of glycerol-3-phosphate (G3P) metabolic genes in adult DRG neurons. We tested five genes including lipin1, *Gpat1*, *Agpat1*, *Agpat3*, *Agpat5*. Gpat, Glycerol-3-phosphate acyltransferase. Agpat, 1-acyl-sn-glycerol-3-phosphate acyltransferase. Adult DRG neurons were dissociated and transfected with the plasmids for 3 days. Neurons were then replated and fixed 24 h after replating. DRG neurites were visualized by using Tuj1 staining. Three mice and 10–20 neurons from each mouse were quantified in each group. ^∗^p ≤ 0.05, ANOVA followed by Dunnett’s test. (B) Representative images of replated neurons from control shRNA and lipin1 shRNA groups with Tuj1 staining. Scale bar: 400 μm. (C) Sections of optic nerves from WT mice at 2 weeks post-injury (WPI). The vitreous body was injected with either AAV-control-shRNA or AAV-lipin1-shRNA. Axons were labeled by CTB-FITC. Scale bar: 100 μm. (D) Number of regenerating axons at indicated distances distal to the lesion site. ^∗∗^p ≤ 0.01, ANOVA followed by Bonferroni’s test, n = 6 mice. (E) Sections of optic nerves from Rosa26-Cas9 mice at 2 WPI injected with either AAV-control-sgRNA or AAV-lipin1-sgRNA. Scale bar: 100 μm. (F) Number of regenerating axons at indicated distances from the lesion site. ^∗∗^p ≤ 0.01, ANOVA followed by Bonferroni’s test, n = 6 mice. (G) Sections of optic nerves from WT mice at 2 WPI injected with AAV-CNTF combined with either AAV-control or lipin1-shRNA. Scale bar: 400 μm. Zoomed-in images are shown in the bottom panel. (G′) Zoomed-in images are shown in the bottom panel. Scale bar: 400 μm. (G″) Zoomed-in images of optic chiasm from (G). Arrows indicate regenerating axons in optic chiasm. Scale bar: 200 μm. (H) Number of regenerating axons at indicated distances distal to the lesion site. ^∗∗^p ≤ 0.01, ^∗^p ≤ 0.05, ANOVA followed by Bonferroni’s test, n = 6 mice. Error bars indicate SEM. See also Figure S1.

To investigate the role of neuronal lipid metabolism in axon regrowth, we systematically knocked down essential genes individually using short hairpin RNA (shRNA) in cultured adult dorsal root ganglion (DRG) neurons (Weng et al., 2018) (Figure S1A). We tested candidates regulating the fatty acid metabolic process, cholesterol synthesis, and glycerol phosphate pathway. Fatty acids in the brain come from fatty acid uptake and synthesis. Fatty acid translocase (CD36) transports long-chain fatty acids through plasma membrane and has relatively high expression level in the brain (Husemann et al., 2002). The rate-limiting enzymes of fatty acid synthesis are acetyl CoA carboxylases (ACC1 and ACC2) (Wakil, 1989). In the cholesterol synthesis pathway, hydroxymethylglutaryl-CoA synthase (HMGCS) is the most upstream enzyme. It catalyzes the reaction from acetyl CoA to hydroxymethylglutaryl-CoA (HMG-CoA) (Bloch, 1965, Bloch, 1992). The rate-limiting and reversible step in cholesterol synthesis is the conversion of HMG-CoA to mevalonate by HMG-CoA reductase (HMGCR) (Bloch, 1965, Bloch, 1992). Another critical gene in the pathway is squalene synthase (FDFT1). A previous report has shown that Fdft1 is required for the neural development (Tozawa et al., 1999). In the glycerol-3-phosphate pathway, glycerol3-phosphate acyltransferase (GPAT) first converts glycerol-3-phosphate to lysophosphatidic acid. Then 1-acylglycerol-3- phosphate acyltransferase (AGPAT) catalyzes the conversion from lysophosphatidic acid to phosphatidic acid. In the following reaction, lipin converts phosphatidic acid to DG (Weiss et al., 1960). We found that most shRNAs did not affect axon regrowth *in vitro* (Figure S1B). Only lipin1 shRNA enhanced axon elongation by 20% (Figures 1A and 1B). Then, we used the optic nerve injury model to assess the effect *in vivo* as previously described (Leon et al., 2000) (Figure S1A). We injected adeno-associated virus (AAV) carrying lipin1 shRNA (shLipin1) into the eyes of adult wild-type (WT) mice to knock down lipin1 in RGCs (Figure S1C). Then, we performed optic nerve crush and examined axon regeneration 2 weeks later. The RGC survival rates were comparable between the two groups (Figures S1D and S1E). Cholera toxin β subunit (CTB) labeling of the optic nerves showed significantly more regenerated axons in mice injected with AAV-shLipin1 than in mice injected with scrambled shRNA (shCtrl) (Figures 1C and 1D), suggesting that lipin1 plays an inhibitory role in axon regeneration *in vivo*.

We next utilized the CRISPR technique to knock out lipin1 in RGCs and assessed the effect. We designed single-guide RNAs (sgRNAs) targeting lipin1 and CRISPR-induced genome editing was verified in Neuro2A cells (Figure S1F). We injected AAV-expressing sgRNAs targeting lipin1 (sgLipin1) with mCherry tag into the eyes of mice constitutively expressing the Cas9 enzyme (Platt et al., 2014). The efficiency of in-vivo gene editing was validated by qRT-PCR using fluorescence-activated cell sorting (FACS)-isolated mCherry-positive retinal cells (Table S2). We found that AAV-sgLipin1 but not AAV-expressing sgRNA targeting LacZ (sgCtrl) promoted axon regeneration after injection into Cas9 mice (Figures 1E and 1F). AAV-sgLipin1 knocked out lipin1 in approximately 50% of RGCs (Figures S1G and S1H). Consistent with the shRNA experiment, RGC survival was not affected by lipin1 knockout (KO) (Figure S1I). We asked whether lipin2, another member of the lipin protein family, plays a role in the axon regeneration. By doing qRT-PCR in sorted RGCs, we showed that lipin2 mRNA was not significantly changed after lipin1 knockdown (KD) (Figure S1J). Then, we used CRISPR to knock out lipin2 in RGCs and assessed the growth effect after optic nerve crush. We found that lipin2 KO did not promote significant regrowth and did not further enhance axon regeneration induced by lipin1 KD (Figures S1K and S1L). Our results suggest that lipin2 does not compensate for the loss of lipin1 in mediating axon regeneration. Furthermore, combining AAV-shLipin1 with AAV expressing ciliary neurotrophic factor (AAV-CNTF) (Pernet et al., 2013) achieved much more robust growth (Figures 1G and 1H). Some axons even reached the optic chiasm within 2 weeks, which was rare after either treatment alone. Lipin1 KD may accelerate the speed of CNTF-induced regeneration. Lipin1 KD also enhanced the axon regeneration induced by Pten KO (Figures S1M and S1N). To examine whether the extent of lipin1 KD is correlated with the extent of axon regeneration, we made another AAV-expressing lipin1 shRNA2, different from the lipin1 shRNA we used above. Lipin1-shRNA2 showed less KD efficiency compared to the lipin1-shRNA (Figure S1O). The axon regeneration induced by AAV-lipin1-shRNA2 was also consistently less (Figure S1P). Thus, through two approaches *in vivo*, we demonstrated that neuronal lipin1 functions as an intrinsic suppressor of axon regeneration.

### Lipin1 Is Selectively Regulated by Aging and Injury in RGCs

**Figure 2:**
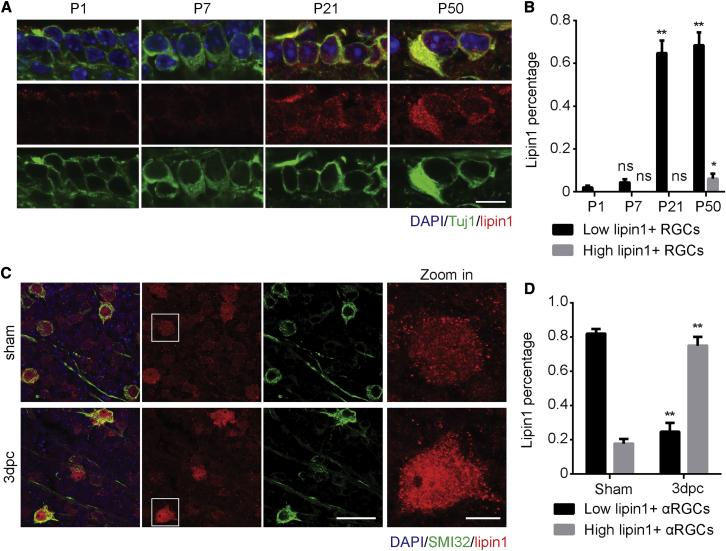
Lipin1 Levels Are Upregulated during Development and after Axotomy (A) Retinal sections from WT mice of different ages (1, 7, 21, and 50 days postnatal) were collected and stained with DAPI (blue), Tuj1 (green), and lipin1 (red). Scale bar: 10 μm. (B) Percentage of RGCs with a low or high lipin1 level at the indicated ages. ^∗∗^p ≤ 0.01, ^∗^p ≤ 0.05, ns, not significant, ANOVA followed by Tukey’s test. (C) Whole-mount retinas from WT mice 3 days after axotomy or sham surgery were collected and stained for DAPI (blue), SMI32 (green), and lipin1 (red). Scale bar: 50 μm. Zoomed-in images are shown in the right panel. Scale bar: 10 μm. (D) Percentage of αRGCs with a low or high lipin1 level indicated by lipin1 staining. ^∗∗^p ≤ 0.01, ANOVA followed by Bonferroni’s test. Error bars indicate SEM. See also Figure S2.

Both aging and response to injury may mediate the intrinsic growth decline of CNS neurons (Belin et al., 2015, Byrne et al., 2014, Cho et al., 2015, Goldberg et al., 2002). We postulated that lipid metabolism could be involved in the decline and examined lipin1 expression in RGCs at different ages and after optic nerve injury. By performing immunostaining, we found that lipin1 protein in RGCs could hardly be detected in young mice but was elevated in adults (Figures 2A and 2B), suggesting that maturation may upregulate lipin1. Interestingly, the vast majority of the αRGCs marked by the SMI32 antibody were lipin1^+^. Over 80% of αRGCs expressed a lower level of lipin1 (low lipin1^+^), while the rest had a high expression level (high lipin1^+^). After optic nerve crush, the percentage of high lipin1^+^ αRGCs increased over 3-fold at 3 days post crush (dpc) (Figures 2C and 2D). In contrast, we did not detect an evident change in the lipin1 level in M1–M3 intrinsically photosensitive RGCs (ipRGCs) by using *Opn4*-GFP mice (Figures S2A and S2B) (Li et al., 2016) or RGCs labeled by TBR2 antibody (Figures S2C and S2D). *Tbr2* is expressed in a subset of RGC types that project to non-image-forming areas (Mao et al., 2014, Sweeney et al., 2014). Thus, maturation and axonal injury selectively regulate lipin1 levels in RGCs

Based on the results, we examined whether the selective regulation of lipin1 in injured αRGCs correlates with axon regeneration. αRGCs have been shown to regenerate their axons after Pten KO (Duan et al., 2015). To identify RGCs with axon regeneration induced by lipin1 KD, we injected fluorogold (FG) into the optic nerve distal to the lesion site. Over 89% of the FG-labeled RGCs were αRGCs with SMI32 staining (Figure S2E). Furthermore, we found that lipin1 elevation in injured αRGCs was suppressed by either deleting Pten in RGCs or overexpressing AAV-CNTF in the retina (Figures S2F and S2G), consistent with the notion that lipin1 functions as an intrinsic inhibitor for RGCs to regenerate the axons.

### Lipin1 Suppresses Growth by Its Phosphatidate Phosphatase Activity

**Figure 3:**
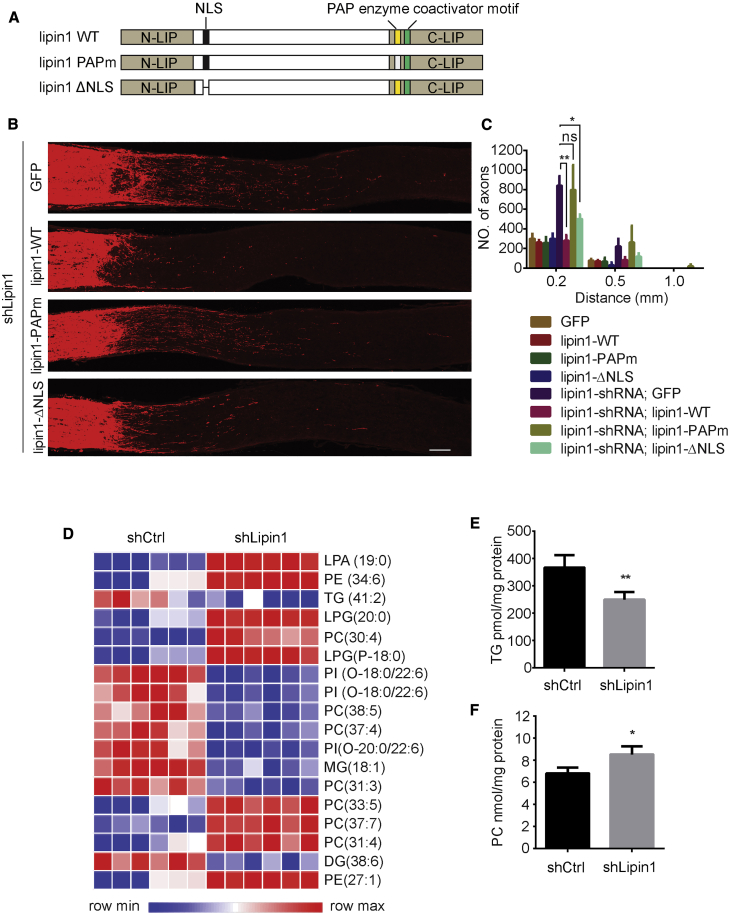
Lipin1 Inhibits Axon Regeneration through Its Phosphatidate Phosphatase Activity and Regulates Glycerolipid Metabolism in Neurons (A) Schematic representation of the different lipin1 overexpression constructs used for the subsequent experiments. (B) Sections of optic nerves from WT mice at 2 WPI, injected with AAV-lipin1-shRNA combined with AAV-GFP, AAV-lipin1-WT, AAV-lipin1-PAPm, or AAV-lipin1-ΔNLS. Scale bar: 100 μm. (C) Number of regenerating axons at different distances distal to the lesion site. ^∗∗^p ≤ 0.01, ^∗^p ≤ 0.05, ANOVA followed by Tukey’s test, n = 5–6 mice. (D) Heatmap represents the alteration of lipidomes after lipin1 KD in cortical neurons. Lipid species with the top 20 VIP are listed. Colors correspond to differences in relative abundance. (E and F) Total TG (E) and PC (F) levels in cortical neurons after AAV-control or lipin1-shRNA treatment. ^∗∗^p ≤ 0.01, ^∗^p ≤ 0.05, Student’s t test. Error bars indicate SEM. See also Figure S3.

Lipin1 has both PAP and transcription coactivator functions (Finck et al., 2006), and we tested whether both functions were involved in lipin1-dependent growth. The PAP catalytic motif (DxDxT) is present in the C-LIP domain of lipin1, and conversion of the first or second aspartate residue in the DxDxT motif to glutamate completely abolishes PAP activity (Finck et al., 2006). The coactivator function can be decoupled from PAP function because mutations in the DxDxT motif abolish PAP activity but do not affect coactivator function (Finck et al., 2006). Thus, through AAVs, we expressed human WT lipin1 (lipin1-WT), lipin1 with a PAP catalytic motif mutation (lipin1-PAPm), and lipin1 lacking a nuclear localization signal (lipin1-ΔNLS) in RGCs with lipin1 depletion (Figure 3A; Figure S3A). We designed shRNA to specifically KD mouse lipin1 and spare exogenous human lipin1. Lipin1-WT but not lipin1-PAPm suppressed axon regeneration to the level of control (Figures 3B and 3C), indicating that PAP activity rather than transcription coactivator function was essential. The result also confirmed that lipin1 shRNA-induced regeneration was not due to potential off-target effects. Consistently, lipin1-ΔNLS also significantly inhibited the growth effect caused by lipin1 depletion (Figures 3B and 3C), suggesting that its nuclear function was not required. RGC survival was not significantly affected in the different groups (Figure S3B). To examine the rescue effect in isolated neurons, we also performed an experiment using adult DRG culture. Lipin1-WT and lipin1-ΔNLS but not lipin1-PAPm inhibited axon elongation in neurons induced by lipin1 KD (Figure S3C), consistent with our *in vivo* results. Thus, through both *in vitro* and *in vivo* experiments, we were able to conclude that the PAP activity of lipin1 plays a major role in inhibiting axon regeneration.

### Lipin1 Regulates Levels of TGs and PLs in Neurons

Because PAP activity is critical for lipin1-dependent axon growth, we hypothesized that lipin1 may control axon regrowth by regulating glycerolipid synthesis in neurons. In budding yeast and mammalian metabolic cells including adipocytes and hepatocytes, lipin1, as a PAP enzyme, plays a major role in lipid homeostasis, especially in the balanced synthesis of TGs and PLs (Pascual and Carman, 2013, Siniossoglou, 2013, Zhang and Reue, 2017). Whether this lipid homeostasis is similarly regulated in neurons is not known. We tested the hypothesis *in vitro*. AAV-shLipin1 or AAV-shCtrl was added to cultured cortical neurons to achieve high KD efficacy (Table S1). We directly measured glycerolipid levels after eliminating glial cells by adding cytosine arabinoside (AraC) to the culture (Figure S3D). To assess the change in the lipid profile in neurons after lipin1 KD, we cultured E18 cortical neurons with AAV-shCtrl or AAV-shLipin1. Lipid extraction of cortical neurons was analyzed by ultra-performance liquid chromatography-mass spectrometry (UPLC-MS) system. We employed a non-targeted approach to identify all the molecules that differ between the two groups. Data were then analyzed by orthogonal partial least-squares discriminant analysis (OPLS-DA) (Bylesjo et al., 2006). Among all the differential molecules, 57 molecules were identified as differential lipid species (p < 0.05 and variable importance for projection [VIP] >1) using lipid databases (Figure 3D). DGs/TGs and PLs comprised 26% and 40% of all the differential lipids, respectively, showing that the two metabolic pathways were highly regulated by lipin1 in neurons. After lipin1 depletion, the levels of TG(41:2) and TG(48:0), which were the most abundant among the eight identified TGs, decreased 18% and 28%, respectively (Figure S3E). The levels of the phosphatidylcholine (PC) lipids PC(30:4) and PC(33:5), which were the most abundant among the nine identified PCs, increased 17% and 15%, respectively (Figure S3F). The levels of the phosphatidylethanolamine (PE) lipids PE(36:4) and PE(37:3), which were the most abundant among the four identified PEs, increased 110% and 151%, respectively (Figure S3G). Cholesterol and free fatty acid were not significantly affected (Figure S3H). Because UPLC-MS might not identify all lipid molecular species, we further measured the total TG and PC levels by performing enzymatic hydrolysis assays. We found that TG levels were decreased by 40% after lipin1 depletion (Figure 3E). Interestingly, PC levels were elevated by 26% (Figure 3F). Thus, the two lipid assay methods consistently demonstrated that, upon lipin1 depletion, storage lipids were lower and membrane lipids were higher in neurons. Because injury triggers lipin1 elevation in RGCs, the data on lipid changes after lipin1 KD indicate that axotomy may program lipid metabolism to increase TG and decrease PL production. This injury-triggered bias in lipid synthesis may contribute to the declined axon growth in CNS neurons. By depleting lipin1, injured neurons may redirect two arms of the branch and shift lipid storage to membrane lipid production for axon regrowth.

### Increasing TG Storage Blocks Axon Regeneration

**Figure 4:**
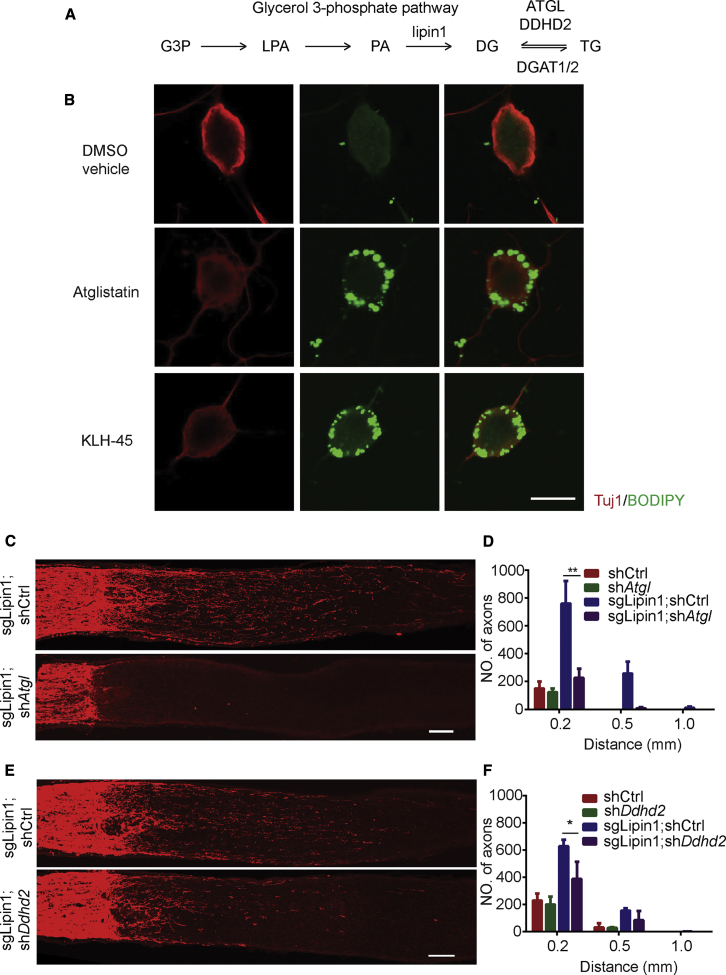
TG Hydrolysis Is Required for the Axon Regeneration Induced by lipin1 Depletion (A) Schematic showing the TG metabolism pathway in mammals. (B) Representative images of DRG neurons cultured with DMSO vehicle, Atglistatin, or KLH-45 for 3 days. BODIPY (green) staining was used to visualize lipid droplet distribution in neurons. Scale bar: 20 μm. (C) Sections of optic nerves from Rosa26-Cas9 mice with lipin1-sgRNA injection at 2 WPI combined with AAV-control or *Atgl* shRNA. Scale bar: 100 μm. (D) Number of regenerating axons at different distances distal to the lesion site. ^∗∗^p ≤ 0.01, ANOVA followed by Tukey’s test, n = 5–6 mice. (E) Sections of optic nerves from Rosa26-Cas9 mice with lipin1-sgRNA injection at 2 WPI combined with AAV-control or *Ddhd2* shRNA. Scale bar: 100 μm. (F) Number of regenerating axons at different distances distal to the lesion site. p ≤ 0.05, ANOVA followed by Tukey’s test, n = 5–6 mice. Error bars indicate SEM. See also Figure S4.

Given that lipin1 regulates the amount of TG in neurons, we asked whether neuronal TG metabolism was important for axon growth induced by lipin1 depletion. TGs are often stored in lipid droplets, which can rarely be detected in neurons. The TG level in the brain is usually much lower than in other tissues (Csaki et al., 2013). Two TG lipases, adipose TG lipase (ATGL) and DDHD2, are active in the brain (Etschmaier et al., 2011, Inloes et al., 2014), and they hydrolyze TGs to DGs and fatty acids (Figure 4A). Knocking out *Ddhd2* in mice causes large amounts of TGs to accumulate in the brain and lipid droplets to form in neurons (Inloes et al., 2014). Treating mice with a specific DDHD2 inhibitor elevates brain TGs within a few days (Inloes et al., 2014), indicating active TG hydrolysis in adult neurons. We first performed experiments to manipulate TG lipases *in vitro*. In cultured adult DRG neurons with vehicle treatment, we could barely detect any lipid droplets (Figure 4B), consistent with the notion that neurons constantly turn over TGs with minimum storage. Either the ATGL inhibitor Atglistatin or the DDHD2 inhibitor KLH-45 dramatically increased TG storage in neurons, as shown by lipid droplet formation (Figure 4B), indicating that these inhibitors effectively targeted TG lipases. Then, we examined the function of TG lipases in axon regeneration *in vivo*. We made AAVs carrying either *Atgl* shRNA (AAV-sh*Atgl*) or *Ddhd2* shRNA (AAV-sh*Ddhd2*) and verified the KD efficacy *in vitro* (Figures S4A–S4D). Then, we injected these AAVs into the eyes of mice with lipin1 depleted in RGCs and examined the effect 2 weeks after optic nerve crush. Expression of each individual virus and the coefficiency of two viruses were validated by whole-mount retina staining (Figure S4E; Table S3). AAV-sh*Atgl* almost completely blocked axon regeneration (Figures 4C and 4D). AAV-sh*Ddhd2* partially suppressed the regrowth (Figures 4E and 4F). RGC survival was not affected by either shRNA (Figures S4F and S4G). We further examined whether increasing TG synthesis affects axon regeneration more generally. In mice with AAV-CNTF injection into the eyes or *Pten* deletion in RGCs, *Atgl* KD significantly suppressed the axon regeneration of the optic nerves (Figures S4H–S4K). Thus, our data indicate that TG hydrolysis is indispensable to the axon regeneration induced not only by lipin1 depletion but also by CNTF or Pten KO.

### Inhibiting TG Synthesis Promotes Axon Regeneration

**Figure 5:**
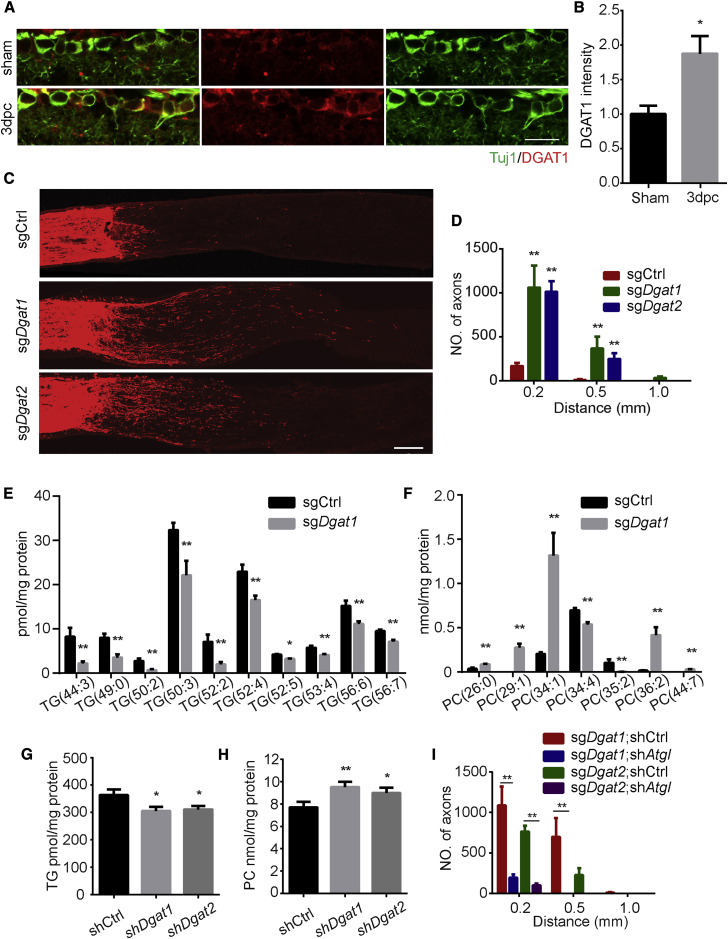
TG Synthesis Inhibition Promotes Axon Regeneration (A) Retinal sections from WT mice 3 days after injury or sham surgery were collected and stained for Tuj1 (green) and DGAT1 (red). Scale bar: 50 μm. (B) Quantification of relative fluorescence intensity of DGAT1 staining in RGCs. ^∗^p ≤ 0.05, Student’s t test, n = 5 mice. (C) Sections of optic nerves from Rosa26-Cas9 mice at 2 WPI. The vitreous body was injected with AAV-control, *Dgat1*, or *Dgat2*-sgRNA. Axons were labeled by CTB-FITC. Scale bar: 100 μm. (D) Numbers of regenerating axons in (C) at indicated distances distal to the lesion site. ^∗∗^p ≤ 0.01, ANOVA followed by Tukey’s test, n = 6 mice. (E and F) Levels of individual TG (E) and PC (F) species normalized to the total protein from either Ctrl or *Dgat1*-shRNA group. The molecular species are indicated as the total number of carbons: the number of double bonds. ^∗∗^p ≤ 0.01, ^∗^p ≤ 0.05, t test, n = 6. (G and H) Levels of total TGs (G) or PCs (H) normalized to the total protein from either Ctrl or *Dgat1*-shRNA group. ^∗∗^p ≤ 0.01, ^∗^p ≤ 0.05, ANOVA followed by Dunnett’s test, n = 6. (I) Quantification of regenerated axons in injured optic nerves from Rosa26-Cas9 mice injected with AAV-*Dgat1* or *Dgat2*-sgRNA at 2 WPI, combined with AAV-control or *Atgl* shRNA. Shown are numbers of regenerating axons at the indicated distances distal to the lesion site. ^∗∗^p ≤ 0.01, ANOVA followed by Tukey’s test, n = 6 mice. Error bars indicate SEM. See also Figure S5.

Because we showed that TG hydrolysis was required, we assessed whether directly decreasing the level of neuronal TGs by restricting their biosynthesis can promote axon growth. In the final step of the glycerol phosphate pathway, DGAT enzymes catalyze DGs into TGs. To assess the effect of blocking the DGAT enzyme, we first used commercially available DGAT1 inhibitors, A-922500 and pradigastat, in culture. DGAT1 inhibitors enhanced the axon elongation of DRG neurons by 60% (Figures S5A and S5B). Then, we performed a KD experiment by transfecting *Dgat1* shRNA (sh*Dgat1*) or *Dgat2* shRNA (sh*Dgat2*) into DRG neurons. Compared with control shRNA, *Dgat1* and *Dgat2* shRNA both significantly boosted axon growth (Figures S5C and S5D). Because axotomy increases lipin1 expression in RGCs, we wondered whether DGAT enzymes were also regulated by axonal injury. Using the DGAT1 antibody, we found that optic nerve injury elevated the level of DGAT1 in RGCs at 3 dpc (Figures 5A and 5B). Furthermore, we deleted DGATs in RGCs through CRISPR by injecting AAV-sgRNA against *Dgat1* (AAV-sg*Dgat1*) or *Dgat2* (AAV-sg*Dgat2*) into the eyes of Cas9 mice and assessed retinal axon regeneration after optic nerve injury. CRISPR-induced genome editing was verified *in vitro* (Figure S5E). Knocking out either *Dgat1* or *Dgat2* enhanced axon regeneration after injury without affecting RGC survival (Figures 5C, 5D, and S5F). We then assessed the lipid profile in cultured neurons with *Dgat1* KD to evaluate whether DGATs may affect the glycerolipids in neurons. All identified TGs decreased after *Dgat1* KD (Figure 5E). Two abundant PC species PC(29:1) and PC(36:2) increased more than 8-fold compared to the control shRNA group (Figure 5F). We then determined the levels of TGs and PCs using lipid hydrolysis assays. Consistently, depleting DGAT1 or DGAT2 decreased the TG level in neurons (Figure 5G). The PC content was increased (Figure 5H). DGs, as a substrate for the DGAT reaction, are also a substrate for PL synthesis. The glycerol phosphate pathway may also provide DGs as an important precursor for PLs. Our interpretation was that, with lipin1 elevation in injured neurons, neuronal depletion of DGAT1 or DGAT2 might divert DGs to the Kennedy pathway to increase PL synthesis. The DGs may come from PA dephosphorylation and TG hydrolysis. Then, we tested whether TG hydrolysis was required for DGAT-dependent axon regeneration. We injected AAV-sh*Atgl* into the eyes of mice with *Dgat1* or *Dgat2* deleted in RGCs and examined the optic nerve 2 weeks after nerve crush. AAV-sh*Atgl* dramatically suppressed the axon regeneration induced by knocking out either *Dgat* (Figures 5I, S5G, and S5H). The data indicate that inhibiting TG synthesis promotes axon regeneration possibly by providing DGs for PL synthesis through TG hydrolysis. Consistent with the notion that lipin1 and DGAT1/2 are on the same lipid synthesis pathway, combining lipin1 KD and Dgat1 KO did not further promote axon regeneration (Figure S5I).

Our data suggest that both lipin1 and DGATs are important in determining the flux of lipids into TGs or PLs and the subsequent axon regeneration. Thus, axotomy drives up two essential enzymes of the glycerol phosphate pathway in neurons, suggesting the critical involvement of this lipid metabolic pathway in axon regeneration failure after injury.

### PL Biosynthesis Is Crucial for Axon Regeneration

Lipin1 depletion or mutation has been demonstrated to generate net increases in PC in several types of cells (Santos-Rosa et al., 2005, Zhang et al., 2012, Zhang et al., 2014). This result may be counterintuitive because DGs, the product of the PAP reaction, are a direct precursor of PC and PE. Several lines of evidence point to the possibility that a reduction in PAP activity elevates PA levels, and subsequently PA may stimulate PCYT1, a critical enzyme for PC synthesis, leading to an increase in PC production (Craddock et al., 2015, Zhang et al., 2019). As two of the major building blocks of membranes, PC and PE are predicted to be essential during axon regrowth. However, disruption of *Pcyt1b* only generates a weak phenotype in axon branches and does not affect the axon elongation of sympathetic neurons *in vitro* (Strakova et al., 2011). The role of PL synthesis in axon regeneration especially *in vivo* has remained elusive. If lipin1 depletion promotes regeneration by redirecting TG synthesis to PL synthesis in neurons, we reasoned that we must evaluate the function of PL synthesis in axon regrowth more extensively.

**Figure 6:**
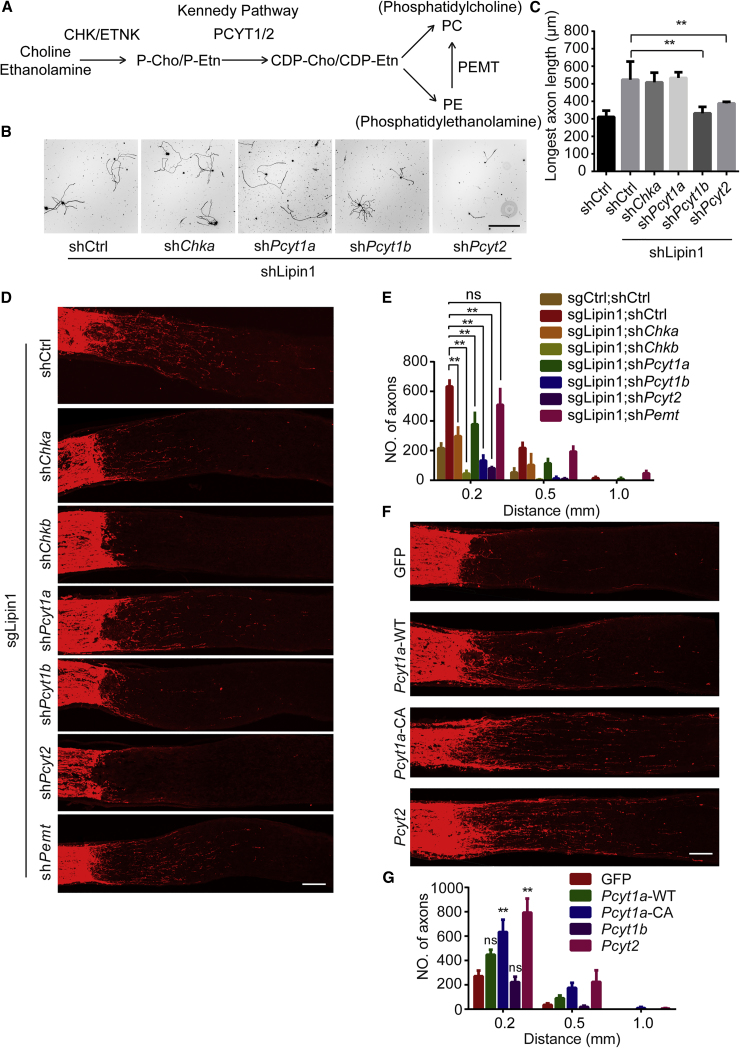
PL Biosynthesis Is Essential for the Axon Regeneration Induced by lipin1 Depletion (A) Schematic showing the PL synthesis pathways in mammals. (B) Representative images of replated neurons from the respective groups with Tuj1 staining. Adult DRG neurons were dissociated and cultured with different AAV shRNA for 10 days. Neurons were then replated and fixed 24 h later. DRG neurites were visualized by Tuj1 staining. Scale bar: 400 μm. (C) Quantification of the length of the longest axon for each DRG neuron in (B). Three mice and 10–20 cells from each mouse were quantified in each group. ^∗∗^p ≤ 0.01, ANOVA followed by Tukey’s test. (D) Sections of optic nerves from Cas9 mice at 2 WPI. The vitreous body was injected with respective AAVs. Axons were labeled by CTB-FITC. Scale bar: 100 μm. (E) Number of regenerating axons at the indicated distances distal to the lesion site. ^∗∗^p ≤ 0.01, ANOVA followed by Tukey’s test, n = 6 mice. (F) Sections of optic nerves from WT mice at 2 WPI. The vitreous body was injected with AAV-GFP, *Pcyt1a*, *Pcyt1a*-CA, or *Pcyt2*. Axons were labeled by CTB-FITC. Scale bar: 100 μm. (G) Number of regenerating axons at the indicated distances distal to the lesion site. ^∗∗^p ≤ 0.01, ANOVA followed by Tukey’s test, n = 6 mice. Error bars indicate SEM. See also Figure S6.

We therefore assessed the role of PL synthesis in axon regeneration induced by lipin1 depletion. We focused on the Kennedy pathway, the major biosynthetic pathway for *de novo* synthesis of PC and PE (Figure 6A) (Gibellini and Smith, 2010). The rate-limiting enzymes in the Kennedy pathway are CTP:phosphocholine cytidylyltransferase α (encoded by *Pcyt1a*), CTP:phosphocholine cytidylyltransferase β (encoded by *Pcyt1b*) for PC, and CTP:phosphoethanolamine cytidylyltransferase (encoded by *Pcyt2*) for PE. Several non-rate-limiting enzymes are also involved in PC synthesis, including *Chka* encoding choline kinase α, *Chkb* encoding choline kinase β, and *Pemt* encoding phosphatidylethanolamine N-methyltransferase. We tested the function of each enzyme in axon regrowth using shRNAs against *Pcyt1a* (sh*Pcyt1*), *Pcyt1b* (sh*Pcyt1b*), *Pcyt2* (sh*Pcyt2*), *Chka* (sh*Chka*), *Chkb* (sh*Chkb*), and *Pemt* (sh*Pemt*) both *in vitro* and *in vivo*. In DRG neurons, none of the tested shRNAs had an evident effect on axon elongation in WT neurons (Figures S6A and S6B). Interestingly, axon growth enhanced by lipin1 shRNA was completely reversed by *Pcyt1b* or *Pcyt2* KD but not by *Pcyt1*a or *Chka* KD (Figures 6B and 6C). In the optic nerve injury model, we did not find a significant effect of any individual shRNA in WT mice (Figure S6C). In mice with lipin1 depletion, *Pcyt1b*, *Pcyt2*, or *Chkb* KD almost completely blocked the enhanced axon regeneration (Figures 6D and 6E). *Pcyt1a* and *Chka* shRNA partially suppressed the regrowth (Figures 6D and 6E), whereas *Pemt* shRNA did not affect regeneration (Figures 6D and 6E). RGC survival was not affected by KD (Figure S6D). In mice with *Dgat1* KO in RGCs, *Pcyt1a*, *Pcyt1b*, *Pcyt2*, or *Chkb* KD significantly inhibited the axon regeneration (Figure S6E). We further examined whether PC synthesis affects axon regeneration more generally. In mice with AAV-CNTF injection into the eyes or *Pten* deletion in RGCs, *Pcyt1b* KD significantly suppressed the axon regeneration of the optic nerves (Figures S6F and S6G). These results suggest that PC and PE synthesis mediated by the Kennedy pathway is indispensable to axon regeneration.

For the gain-of-function experiments, we assessed whether stimulating PL production by overexpressing *Pcyt1a*, *Pcyt1b*, and *Pcyt2* in RGCs promotes axon regeneration. For *Pcyt1a*, we also included constitutively active *Pcyt1* (*Pcyt1a*-CA) by removing the amphipathic C-terminal lipid-binding domain to prevent autoinhibition of the enzyme catalytic activity (Craddock et al., 2015). AAV carrying *Pcyt1a*, *Pcyt1a*-CA, *Pcyt1b*, or *Pcyt2* was injected into the retinas of WT mice (Figure S6H), which were subjected to optic nerve crush. *Pcyt1a*-CA and *Pcyt2* overexpression enhanced axon regeneration at 2 weeks after injury (Figures 6F and 6G). Collectively, our results demonstrate that PL biosynthesis plays an essential role in axon regeneration induced by lipin1 depletion.

We have shown that αRGCs preferentially regenerate their axons after lipin1 KD (Figure S2E). To test the hypothesis that selective lipin1 upregulation in αRGCs after injury may inhibit the axon regeneration by increasing TGs and decreasing PLs, we compared the mRNA levels of *Atgl*, *Pcyt1a*, and *Pcyt1b* in WT αRGCs and M1–M3 ipRGCs. In Opn4-GFP mice with sham or optic nerve injury, we injected micro-Ruby into the optic nerve to label RGCs. Under the fluorescence microscope, we manually isolated RGCs labeled by micro-Ruby or GFP after retina dissociation and did single-cell qRT-PCR. RGCs with high *Spp1* expression were regarded as αRGCs (Duan et al., 2015). GFP was used to mark M1–M3 ipRGCs. We found that *Atgl* and *Pcyt2* were selectively downregulated in αRGC but not in M1–M3 ipRGC (Figure S6I). The results support that the glycerolipid metabolism is selectively regulated in RGCs after injury and mediates the axon regeneration.

### TG Synthesis Inhibition Mediates Peripheral Axon Regeneration

**Figure 7:**
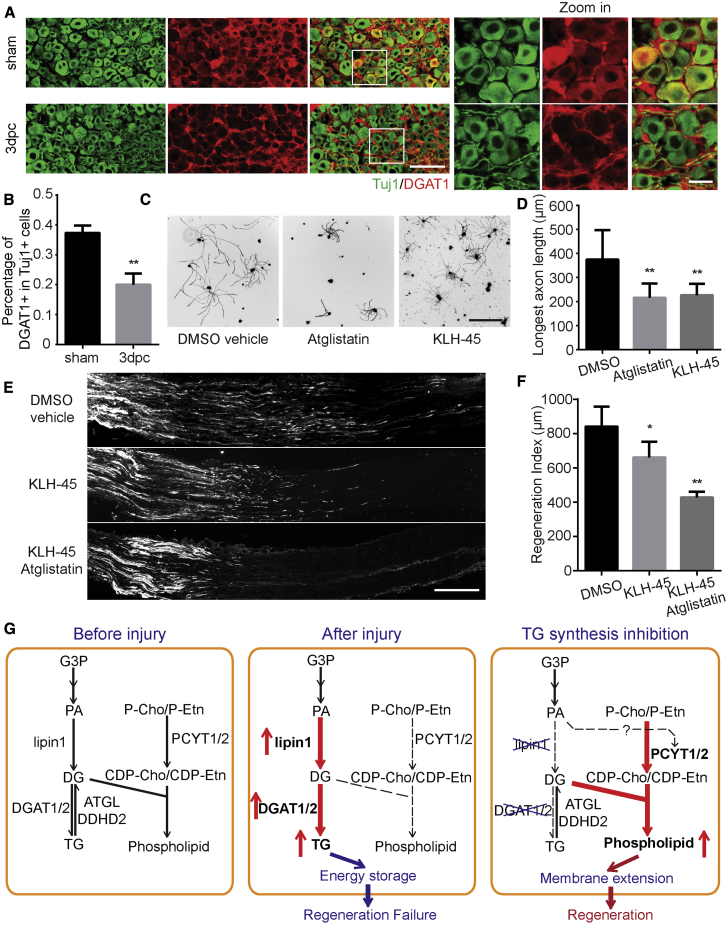
TG Synthesis Inhibition Promotes Spontaneous Peripheral Axon Regeneration (A) DRG sections from WT animals 3 days after sciatic nerve crush or sham surgery, stained with Tuj1 (green) or DGAT1 (red) antibodies. Scale bar: 100 μm. Zoomed-in images are shown in the right panel. Scale bar: 20 μm. (B) Percentage of DGAT1^+^ DRG neurons in (A). ^∗∗^p ≤ 0.01, Student’s t test. (C) Representative images of DRG neurons in primary cultures treated with DMSO vehicle, Atglistatin (10 μM), or KLH-45 (10 μM). DRG neurites were visualized by Tuj1 staining. Scale bar: 400 μm. (D) Quantification of the length of the longest axon for each DRG neuron in (C). Three mice and 10–20 cells from each mouse were quantified in each group. ^∗∗^p ≤ 0.01, ANOVA followed by Dunnett’s test. (E) Sections of sciatic nerves from WT animals treated with DMSO, KLH-45, or KLH-45 combined with Atglistatin. Axons are visualized by SCG10 staining. Scale bar: 400 μm. (F) Quantification of regenerating sensory axons in (E). ^∗∗^p ≤ 0.01, ^∗^p ≤ 0.05, ANOVA followed by Dunnett’s test. (G) A working model of the glycerol phosphate pathway in axon regeneration by using diagrams of glycerolipid metabolism redirection in intact, injured, or regenerating neurons. TG and PL metabolism maintains homeostasis in intact neurons. After CNS axonal injury, lipin1 and DGAT1 upregulation leads to TG accumulation in neurons and eventually inhibits axon regeneration. However, after lipin1 or DGAT1/2 inhibition, TG synthesis gives way to PL synthesis to support axon regeneration. See also Figure S7.

As the PNS neurons spontaneously regenerate their axons and possess a stronger growth capacity than CNS neurons (Chandran et al., 2016), we examined how the glycerol phosphate pathway is regulated in adult DRG neurons *in vivo*. Three days after sciatic nerve injury in adult WT mice, we performed immunostaining to examine the levels of lipin1 and DGAT1 proteins in DRG neurons. Lipin1 protein was detected in most neurons, and the level was maintained after injury (Figure S7A). KD lipin1 in DRG neurons did not significantly enhance the spontaneous axon regeneration at 3 days after sciatic nerve crush (Figures S7B–S7D). DGAT1 was found in both neuronal and non-neuronal cells. In contrast to the injury-induced DGAT1 upregulation observed in RGCs, the level of neuronal DGAT1 was significantly decreased at 3 days after injury (Figure 7A). The percentage of DGAT1^+^ DRG neurons was reduced by ∼50% compared with sham control (Figure 7B). The staining of non-neuronal cells did not change obviously. This downregulation was not merely correlative because we have already shown that both DGAT1 inhibitors and KD can enhance DRG axon elongation *in vitro* (Figures S5A–S5D). Because our hypothesis was that DGAT1 downregulation inhibits TG synthesis and directs TG-derived DGs to PL synthesis, we further examined the role of TG hydrolysis in DRG axon regeneration *in vitro* and *in vivo*. In dissociated primary DRG neuron culture, TG lipase inhibitors—Atglistatin and KLH-45—significantly inhibited axon elongation (Figures 7C and 7D). Consistently, *Ddhd2* KD in isolated DRG neurons suppressed axon elongation in culture, and as a positive control lipin1 KD increased the axon length (Figure S7E). Then, we systematically administered vehicle, KLH-45, or KLH-45 combined with Atglistatin into WT mice, crushed the sciatic nerve, and allowed the sensory axons to regrow for 2 days before examination (Figure S7F). SCG10 was used as a marker to specifically label the regenerated sensory axons in the sciatic nerve as previously described (Chen et al., 2017, Shin et al., 2014). In the control mice, sensory axons robustly regenerated several hundred micrometers within 2 days. This spontaneous regeneration was modestly inhibited by KLH-45 alone and markedly suppressed by the combination of KLH-45 and Atglistatin (Figures 7E and 7F). Neuronal survival was not affected by the compounds (Figure S7G). The results demonstrate that TG hydrolysis is required for peripheral axon regeneration. The differential regulation of the glycerol phosphate pathway in injured PNS and CNS neurons may contribute to the different regenerative capabilities.

## Discussion

Our study reveals a critical role of the neuronal glycerolipid biosynthesis pathway in response to injuries and axon regeneration. Previous studies have demonstrated that successful regeneration in neurons requires activation of proregenerative transcription and translation (Cho et al., 2015, Moore et al., 2009, Park et al., 2008, Sun et al., 2011, Terenzio et al., 2018), epigenetic regulation (Cho et al., 2013, Gaub et al., 2010, Oh et al., 2018, Rivieccio et al., 2009, Weng et al., 2017, Weng et al., 2018), cytoskeletal dynamics and transport (Blanquie and Bradke, 2018, Hellal et al., 2011, Nawabi et al., 2015), mitochondrial mobility and localization (Cartoni et al., 2016, Luo et al., 2016, Zhou et al., 2016), among other processes. Our findings indicate that neuronal lipid metabolism also needs to be properly coordinated for injured axons to regenerate. We propose a model in which axotomy disrupts the homeostatic synthesis of glycerolipids in the glycerol phosphate pathway by increasing lipin1 and DGAT1 and limits axon regeneration in CNS neurons by directing the lipid flux toward energy storage rather than membrane extension (Figure 7G). Forced lipin1 depletion reduces the supply for TG production on the one hand and upregulates PA on the other hand, which may stimulate PCYT1 activity and subsequent PL synthesis. In addition, active TG hydrolysis generates a supply of DGs as the precursor for PLs. DGAT1 depletion may suppress TG production and drive DGs to the Kennedy pathway, a strategy adopted by peripheral neurons for axon regeneration. It is also possible that released free fatty acids from TG hydrolysis may also contribute to the lipid precursor supply through early reaction steps of the glycerol 3-phosphate pathway. Taken together, our model stresses that the balanced synthesis between TGs for storing energy and PLs for building membranes may determine the axon regeneration competence.

Several lines of evidence support our working hypotheses. First, lipin1 depletion promoted axon regeneration after optic nerve crush and decreased TGs while increasing PLs in neurons. Directly suppressing *de novo* synthesis of TG by KD *Dgat* also boosted axon growth and the PL level. Second, the changes in TG and PL levels were more than correlation because either forcing TG storage or inhibiting PL synthesis enzymes almost completely blocked lipin1- and Dgat1-dependent axon regeneration *in vivo*. Third, lipin1 level was reduced in regenerating RGCs induced by either Pten KO or CNTF, two independent mechanisms mediating axon regeneration (Sun et al., 2011). Fourth, either *Atgl* KD or *Pcyt1b* KD suppressed Pten KO and CNTF-induced axon regeneration. Fifth, in PNS neurons that spontaneously regenerate their axons, DGAT1 was downregulated upon axotomy, suggesting that the glycerol phosphate pathway is actively involved in peripheral axon regeneration by shifting lipid storage toward membrane lipid production. Indeed, TG hydrolysis is required for sensory axon regeneration after sciatic nerve crush, indicating that this lipid metabolic pathway affects adult axon regeneration more broadly.

In the plant, *Arabidopsis thaliana*, double mutation of pah1 and pah2 (homologs of mammalian lipin) increases the level of PLs with massive membrane expansion via increased transcription of several PL synthesis genes (Craddock et al., 2015). Studies in yeast show that loss of smp2 (a homolog of mammalian lipin) promotes the transcription of PL synthesis genes and leads to nuclear membrane expansion (Santos-Rosa et al., 2005). In addition, depleting the PAP activity in rodent enterocytes increases the PC level by increasing PCYT1A protein. It is likely that diminished PAP activity induces PA accumulation, which enhances PCYT1A and possibly other enzymes (Zhang et al., 2019). In mouse neurons, lipin1 depletion likely promotes PL synthesis through a similar mechanism.

The glycerol phosphate pathway regulates the synthesis of glycerolipids at different growth stages or upon stress. In yeast, membrane lipids are preferentially synthesized from the precursor PA during exponential growth. When cells progress to the stationary phase upon nutrients exhaustion, PA is directed toward TG synthesis. In metabolic cells, the TG represents the major neutral lipids stored in cells and excessively synthesized TGs mainly exist in lipid droplets. The incorporation of synthesized TGs into lipid droplets is a protective mechanism under certain stress conditions. This mechanism can prevent the accumulation of unesterified lipids that may trigger inflammatory responses and cause lipotoxicity in cells. In our study, axonal injury enhanced lipin1 levels in RGCs, which may have increased TG synthesis. This could be a protective response from RGCs, although it is unclear whether any neuronal lipid droplets were formed. It is challenging to identify neuronal LDs that are rare or transient. Interestingly, in axotomized adult rabbit vagal neurons that regenerate their axons poorly, lipid accumulation can be detected by electron microscopy (Aldskogius, 1978). In other cases, lipid droplets can be detected in axons of *Aplysia*, neurons in the Huntington’s disease model, and cortical neurons in culture (Welte, 2015). In the adult mouse brain, LDs are rarely found. But they can accumulate inside neurons of *Ddhd2* KO mice and also in adult DRG neurons when TG lipase inhibitors were added in culture as shown in our study. The physiological role of constant and quick turnover of TG inside adult neurons is not entirely clear. Our data suggest that it may be related to supplying membrane lipids. Under injury conditions, it may contribute to rebuild axons, whereas, under naive conditions, it may be involved in supplying membrane structures such as various vesicles, crucial for maintaining normal neuronal functions. However, we cannot exclude the possibility that other functions of PLs may contribute to the growth effect. Further studies will be necessary to elucidate the functional role of TG hydrolysis in neurons.

The endoplasmic reticulum (ER) is the largest organelle and forms a continuous network throughout the neuron including the axon (González et al., 2016, Wu et al., 2017). The ER membrane is a major site of lipid biosynthesis, including TG, PC, and PE synthesis, and houses many of the enzymes involved (Zhang and Reue, 2017). Axonal injury inevitably causes damages to the tubular ER in the axons. In addition, optic nerve injury induces ER stress in RGCs (Hu et al., 2012). Previous studies suggest that the expression of one of the lipin family members, lipin2, is induced by ER stress in liver cells (Ryu et al., 2011). Thus, axotomy-induced ER stress in neurons may also affect lipid synthesis through regulating lipin. This warrants a future study on the relationship between axon injury, ER stress, and lipid metabolic changes.

Lipin1 mutant mice have lipodystrophy with significant reduction in fat mass and other pathological defects, which makes lipin1 a non-ideal translational target. DGAT2 is essential for survival in mice (Stone et al., 2004). However, *Dgat1* KO mice are viable, generally lean, and resistant to diet-induced obesity (Smith et al., 2000). DGAT1 has emerged as an attractive druggable target for certain metabolic disorders (Chen and Farese, 2005, DeVita and Pinto, 2013). Further studies should investigate whether local or systematic inhibition of DGAT1 can achieve a similar growth effect to that of the genetic KO in a clinically relevant injury model such as spinal cord injury. In summary, our results demonstrate a potentially translatable strategy, which involves tailoring glycerolipid synthesis in support of axon regeneration.

## STAR★Methods

### Key Resources Table

**Table:** 

REAGENT or RESOURCE	SOURCE	IDENTIFIER
**Antibodies**		

Rabbit anti-FITC	Invitrogen	Cat# 71-1900; RRID:AB_2533978
Mouse anti-SMI32	BioLegend	Cat# 801701; RRID:AB_2564642
Mouse anti-Tuj1	BioLegend	Cat# 801202; RRID:AB_10063408
Rabbit anti-Tuj1	BioLegend	Cat# 802001; RRID:AB_2564645
Rabbit anti-Lipin1	Santa Cruz	Cat# sc-98450; RRID:AB_2135907
Rabbit anti-ATGL	Cell Signaling	Cat# 2439; RRID:AB_2167953
Rabbit anti-DDHD2	ProteinTech	Cat# 25203-1-AP
Chicken-anti Tbr2	EMD Millipore	Cat# AB15894; RRID:AB_10615604
Mouse anti-HA-tag	Cell Signaling	Cat# 2367; RRID:AB_10691311
Mouse anti-DGAT1	Santa Cruz	Cat# sc-271934; RRID:AB_10649947
Goat anti Mouse 555	Invitrogen	Cat# A-21424; RRID:AB_141780
Goat anti Mouse Cy5	Invitrogen	Cat# A10524; RRID:AB_2534033
Goat anti Rabbit 555	Invitrogen	Cat# A-21429; RRID:AB_2535850
Goat anti Rabbit Cy5	Invitrogen	Cat# A10523; RRID:AB_2534032
Goat anti Chicken 555	Invitrogen	Cat# A-21437; RRID:AB_2535858
Goat anti Chicken 647	Invitrogen	Cat# A-21449; RRID:AB_2535866

**Bacterial and Virus Strains**		

pAAV.hSyn.eGFP.WPRE.bGH	James M. Wilson (unpublished data)	Cat#105539; RRID:Addgene_105539
pAAV.hSyn.HI.eGFP-Cre.WPRE.SV40	Penn Vector	Cat#P1848
pAAV.U6.shRLuc.CMV.EGFP.SV40	Penn Vector	Cat#P1867
pAAV-U6. sgRNA(SapI)_hSyn-GFP-KASH-bGH	Swiech et al., 2015	Cat#60958; RRID:Addgene_60958

**Chemicals, Peptides, and Recombinant Proteins**		

Cholera Toxin B Subunit, FITC Conjugate	Sigma-Aldrich	Cat#C1655
Fluorogold	Invitrogen	Cat#H22845
Papain	Sigma-Aldrich	Cat#P4762
micro-Ruby	Invitrogen	Cat#D7162
Fetal bovine serum	HyClone	Cat#SH3007103
Neurobasal	GIBCO	Cat#10888022
Paraformaldehyde (PFA)	Sigma-Aldrich	Cat#30525-89-4
Optimal Cutting Temperature compound	SAKURA	Cat#4583
Triton X-100	Sigma-Aldrich	Cat#T8787
Normal goat serum	Invitrogen	Cat#50062Z
DMSO	Sigma-Aldrich	Cat#D2650
Sucrose	Invitrogen	Cat#15503022
DAPI	Sigma-Aldrich	Cat#D9542
Atglistatin	MedChemExpress	Cat#HY-15859
KLH-45	Sigma-Aldrich	Cat#SML1998
A-922500	Sigma-Aldrich	Cat#A1737
Pradigastat	MedChemExpress	Cat#HY-16278
BODIPY	Sigma-Aldrich	Cat#793728
Collagenase	Roche	Cat#11088858001
Laminin	GIBCO	Cat#23017015
PhosSTOP	Roche	Cat#04906837001
cOmplete Tablets	Roche	Cat#05892791001

**Critical Commercial Assays**		

Mouse Neuron Nucleofector Kit	Lonza	Cat#VPG-1001
PureLink Genomic DNA Mini Kit	Thermo	Cat#K182001
T7 Endonuclease I	NEB	Cat#M0302S
Phosphatidylcholine Assay Kit	Abcam	Cat#ab83377
Triglyceride Assay Kit	Abcam	Cat#ab65336
RNeasy Mini Kit	QIAGEN	Cat#74104
SuperScript II Reverse Transcriptase	Invitrogen	Cat#18064014
KAPA HiFi HotStart ReadyMix	Roche	Cat#KK2601
LightCycler 480 SYBR Green I Master	Roche	Cat#04707516001

**Experimental Models: Cell Lines**		

Neuro2a	ATCC	Cat#CCL-131; RRID:CVCL_0470

**Experimental Models: Organisms/Strains**		

Mouse: Adult C57Bl6/J	Charles River	N/A
Mouse: Opn4-GFP C57Bl6/J	Schmidt et al., 2008	N/A
Mouse: Rosa26-Cas9 knockin C57Bl6/J	Platt et al., 2014	Cat#026179; RRID: IMSR_JAX: 026179
Mouse: Adult Pten^flox^	Park et al., 2008	N/A

**Oligonucleotides**		

shRNA and sgRNA sequences: Table S4	This paper	N/A
PCR primers: Table S5	This paper	N/A

**Recombinant DNA**		

pAAV-hSyn-lipin 1 WT	This paper	N/A
pAAV-hSyn-lipin 1 PAPm	This paper	N/A
pAAV-hSyn-lipin 1 ΔNLS	This paper	N/A
pAAV-hSyn-*Pcyt 1a*	This paper	N/A
pAAV-hSyn-*Pcyt 1b*	This paper	N/A
pAAV-hSyn-*Pcyt 1a-CA*	This paper	N/A
pAAV-hSyn-*Pcyt 2*	This paper	N/A

**Software and Algorithms**		

ImageJ	NIH	https://imagej.nih.gov/ij/
Prism 6	GraphPad	https://www.graphpad.com/
Progenesis QI	Waters-Nonlinear	http://www.nonlinear.com/progenesis/qi/
SPSS Statistics	SPSS Inc.	https://www.ibm.com/hk-en/products/spss-statistics
SIMCA 14.1	Umetrics	https://umetrics.com/

### Lead Contact and Materials Availability

Further information and requests for resources and reagents should be directed to and will be fulfilled by the Lead Contact, Kai Liu (kailiu@ust.hk). All unique/stable reagents generated in this study are available from the Lead Contact without restriction.

### Experimental Model and Subject Details

#### Animals

Wild-type (WT, C57BL/6J, Charles River) mice of both genders at P1, P7, P21 or P50 were used in experiments of examining the lipin1 levels in RGCs by aging. In all the other experiments, wild-type and transgenic mice of both genders (7-8 weeks old) were used as indicated. Constitutive SpCas9 knockin mice (stock number: JAX_026179) (Platt et al., 2014) were obtained from Jackson Laboratories. Opn4-GFP mice (Li et al., 2016) were obtained from the Mutant Mouse Regional Resource Center, an NIH funded strain repository, and the strain was donated by the National Institute of Neurological Disorders and Stroke funded Gene Expression Nervous System Atlas (GENSAT) bacterial artificial chromosome (BAC) transgenic project. *Pten*-floxed mice (Park et al., 2008) were gifts from Dr. Zhigang He (Boston Children’s Hospital). Housing and breeding conditions followed standard procedures. Experimental and control mice were littermates and were kept together before experiments. All experimental procedures were performed in compliance with animal protocols that were approved by the Animal and Plant Care Facility at the Hong Kong University of Science and Technology.

#### Cell lines

Neuro2A cells (ATCC, stock number: CCL-131) were maintained at 37°C under a humidified 5%CO2 atmosphere using Dulbecco Modified Eagle Medium (DMEM) and supplemented with fetal bovine serum (HyClone).To test the KD efficiency of shRNAs, Neuro2A cells were first cultured on a 12-well plate to 70%–80% confluency. Cells were then transfected with 1 μg shRNA plasmid by Lipofectamine 3000 for 48 h. The transfection procedure was performed according to the manufacturer’s protocol.

#### Primary cell cultures

DRG primary culture and replating were performed as previously described (Weng et al., 2018). In brief, for primary culture, L4-L6 DRGs from 7-8 weeks wild-type mice of both genders were dissected and then digested in 0.5% collagenase for 1.5 h. After termination of digestion, DRGs are pipetted 20-30 times in a tube for complete dissociation. Neurobasal-A with B27 as a supplement was used as a medium for DRG culture. Virus was added at 1 day *in vitro* (DIV1) for genetic manipulation. Atglistatin (10 μM), KLH-45 (1 μM), paradigastat (1 μM) or A-922500 (500 nM) treatment was used for ATGL, DDHD2 or DGAT1 inhibition.

For replating DRG neuron culture, at DIV9-11 of primary DRG culture, cells were gently pipetted on culture dishes. Usually, cells were flushed by 20-30 rounds of pipetting in each well of a 6-well plate. After all the cells were resuspended, they were replated onto a 24-well plate. Fixation and staining were performed 24 h after replating. Tuj1 staining was used to visualize axons and cell bodies of neurons. The lengths of the longest neurites from each DRG neuron were measured manually by NeuronJ in ImageJ. Average lengths of 10-20 neurites from 3 individual mice were used in each group.

For the *in vitro* screening, dissociated DRG neurons from 4-6 DRGs were first resuspended in 100 μL Amaxa mouse neuron electroporation buffer (Lonza) containing 5 μg respective plasmids. The cell suspension was then transferred to 2mm cuvette (Lonza) for electroporation. The electroporation was done by using the Amaxa Nucleofector System (Lonza). The primary culture and replating procedures were described as above.

Cortical neurons were isolated from E18 mouse embryo. Briefly, the cortex from E18 C57/B6 mice of both genders was dissected and digested in 0.5 mg/mL papain for 30 min. Then, 100 μL fetal bovine serum (HyClone) was added to inactivate papain. The cells were then placed into Neurobasal medium (GIBCO), supplemented with B27 (GIBCO) and 1% penicillin-streptomycin (10,000 U/mL; GIBCO). AraC (100 nM; Sigma) was used to inhibit glial proliferation in DIV1-DIV3. After 10 days of culture with AAV-scramble, AAV-shLipin1, AAV-sh*Dgat1* or AAV-sh*Dgat2*, cells were harvested, and then TG or PC levels were measured according to the manufacturer’s protocol (ab65336 and ab83377).

### Method Details

#### AAV construct and packaging

AAV serotype 2/1 was used for CNTF overexpression. AAV serotype 2/2 was used for all the other overexpression and shRNA AAVs. The AAV construct backbone for overexpression and shRNAs was obtained from Penn Vector Core. qRT-PCR was used for virus titer measurement. The virus titer was ∼10^13^ GC/mL.

#### Western blot

For western blot analysis, cells were harvested in ice-cold PBS and then lysed in RIPA buffer for 45 min. RIPA buffer consisted of 50 mM Tris·HCl at pH 8.0, 150 mM NaCl, 1% Nonidet P-40, 0.5% Na-deoxycholate, 0.5% SDS supplemented with EDTA-free cOmplete ULTRA tablets (Roche), and PhosSTOP Complete Easypack (Roche). Cell lysates were centrifuged at 16000 g for 10 min. 4x SDS sample buffer was added to the supernatant of cell lysates. Western blotting was performed according to the standard protocol.

#### Genomic DNA extraction and T7E1 assay

To validate the efficiency of sgRNAs, Neuro2A cells were first cultured on a 12-well plate to 70%–80% confluency using Dulbecco Modified Eagle Medium (DMEM) and supplemented with fetal bovine serum (HyClone). Cells were then transfected with 1 μg SpCas9 and respective sgRNA plasmid by Lipofectamine 3000 for 72 h. Genomic DNA of Neuro2A was purified by PureLink Genomic DNA Mini Kit. The amplification of target DNA fragment and efficiency testing of individual sgRNAs were performed with manufacturer’s protocol of NEB T7 Endonuclease I. Primers used for DNA fragment amplification were listed in STAR Methods. In brief, purified PCR product was denatured and then annealed. If indels existed in the PCR product, heteroduplex DNA would form after annealing. Then T7 Endonuclease 1 was added to recognize and cleave theheteroduplex DNA at the mismatching site. Finally, we used gel electrophoresis to analyze the fragments in the PCR product.

#### Optic nerve injury and quantification

Intravitreous injection and optic nerve injury were performed as previously described. Mice were intravitreously injected with AAV at postnatal day 28 (P28)-P42. In brief, mice were anesthetized by a mixture of ketamine (80 mg/kg) and xylazine (10 mg/kg). We clamped the edge of the eyelid with a small artery clamp to expose the conjunctiva. Two microliters of virus was injected gently into each vitreous body using a Hamilton syringe. Meloxicam (1 mg/kg) was injected as analgesia after the operation. Mice with obvious eye inflammation or shrinkage were sacrificed and excluded from further experiments.

Four weeks after virus injection, intraorbital optic nerve crush was performed as previously reported. After the mice were anesthetized and an incision was made on the conjunctiva, the optic nerve was crushed by jeweler’s forceps (Dumont #5; Fine Science Tools) for 2 s at 1-2 mm behind the optic disk. To visualize regenerating axons, RGC axons in the optic nerve were anterogradely labeled by 1.5 μL CTB (2 μg/μL, Invitrogen) 12 days after injury.

Whole-mount Tuj1 staining was used to determine the number of surviving RGCs at two weeks after optic nerve crush. The retina was dissected and stained following the previous protocol. Briefly, the retina was washed with 1X PBS three times in a 24-well plate and then incubated with PBS with 4% normal goat serum (NGS) for 30 mins. After incubation with the Tuj1 antibody overnight at room temperature, the retina was washed with PBS three times and incubated with secondary antibody for 1 h. After the tissue was washed with PBS, the retina was mounted onto glass slides, and images were taken under a confocal microscope (Zeiss, LSM Meta710; 40X and 63X objective). For each retina, 12 images were taken from different quarters, which covered the peripheral and central regions of the retina. An individual who was blind to different groups counted the number of Tuj1+ RGCs.

To quantify the number of CTB-FITC-traced axons after optic nerve crush, the optic nerve was dissected carefully and placed longitudinally for cryo-section (section thickness: t = 8 μm). The serially collected optic nerve tissue was stained with the FITC antibody, mounted onto glass slides and imaged under a confocal microscope (Zeiss, LSM Meta710; 10X objective). Captured images were stitched together by using ImageJ. The images of optic nerve with CTB channel were converted into red and exported. Representative optic nerves were cropped from the stitched images. This process may leave some dashed lines or uneven background around the optic nerve. Five images were taken for each optic nerve. The following formula was used to quantify the number of regenerated axons at different distances from the lesion site: ∑ad = πr^2^ × [average axon numbers/mm]/t. The r is the radius of the optic nerve at the counting site, and the average axon numbers/mm were determined by the average numbers of (axon numbers) / (nerve width at the counting site) of the five sections. The t is the section thickness (8 μm). Axon numbers were counted by an individual who was blind to different groups.

#### Retrograde Labeling of Regenerating RGCs

At thirteen days after the optic nerve crush, mice were anesthetized and placed in a stereotaxic holder. The crushed optic nerve was gently exposed, with a pulled-glass micropipette attached to a Hamilton syringe, we slowly injected FG (100 nL, 5% wt/vol) into the optic nerve ∼2 mm distal to the lesion site. 1 day later, the animals were sacrificed and the retinas were dissected for staining.

#### RGC isolation and qRT-PCR

For isolating single RGCs by mouth-pipetting, 8 weeks old Opn4-GFP mice received an optic nerve crush or sham injury, then micro-Ruby (500 nL, 5% wt/vol. Invitrogen) was slowly injected into the optic nerve. 3 days later, the animals were sacrificed and the retinas were dissected and digested in 0.5 mg/mL papain for 35 min. Then, fetal bovine serum (HyClone) was added to stop the digestion. After centrifugation, the cells were then suspended into Neurobasal medium for further dissociated into single cell suspensions. With a mouth pipette, GFP positive cells or micro-Ruby positive (red) cells were gently pipetted into a new medium drop, after several times repeating, one cell was pipetting into a tube containing lysis buffer. The cell lysis, RT-PCR and pre-amplification were performed with previously described smart-seq2 protocol (Picelli et al., 2014). Pre-amplified cDNA was used as templates for qRT-PCR.

For isolating GFP or mCherry positive RGCs by FACS, the dissociation was done by the above procedure. Cell sorting was performed with BD FACSAria III instrument. Dissociated retinal cells were separated based on size (forward scatter) and surface characteristics (side scatter) as well as viability (DAPI staining). Doublets or clots were excluded based on the FSC-H-versus-FSC-A ratio. Retinal cells from control mice without any virus injection were used to set up gates for each experiment. 5000 sorted cells were collected in each replicate and RNA was extracted by RNeasy Mini Kit (QIAGEN). Total RNA was reverse transcribed to cDNA by SuperScript II Reverse Transcriptase using manufacturer’s protocol.

For qRT-PCR, each sample was run in 2-4 replicates. *Gapdh* was used as endogenous control. The qRT-PCR was done by manufacturer’s protocol of LightCycler 480 SYBR Green I Master.

#### Intrathecal injection of AAVs

AAV1-control or lipin1 shRNA was injected to thecal sac between L5 and L6. Briefly, mice were anesthetized with a mixture of ketamine (80 mg/kg) and xylazine (10 mg/kg). An incision was conducted in the middle line. Then dura was exposed by laminectomy. A microforged glass needle was inserted into the median area and 3 μL virus was slowly infused to the spinal cord. The skin was sutured with stainless clip and the mice were placed on heating pad until awake. The sciatic nerve crush was done 4 weeks after injection.

#### Sciatic nerve injury and quantification

KLH-45 (30mg/kg/day) or Atglistatin (40mg/kg/day) were delivered by intraperitoneal injection for consecutive 5 days before injury and 2 days after injury. Same dosage of DMSO injection was used as control treatment. Sciatic nerve injury was performed as previously described. Briefly, after an incision was made on the skin at the middle thigh level, muscle was gently dissected to expose the sciatic nerve. Then, the sciatic nerve was crushed for 10 s by forceps (Dumont #2; Fine Science Tools). For the sham group, the sciatic nerve was only exposed but not crushed.

Sciatic nerve sections with SCG10 staining were used to quantify the regeneration index. A column with a width of 50 pixels was drawn at different distances from the lesion center, and the average intensity of SCG10 staining was measured using ImageJ. The distance between the lesion center and the column with half the intensity of the lesion center was considered as the regeneration index.

#### Immunohistochemistry

For BODIPY staining on cultured DRG neurons, cells were first fixed in 4% PFA for 10 min and permeabilized with 0.1% Triton X-100 in 4% NGS. After cells were blocked, the Tuj1 antibody was applied in blocking buffer and incubated at 4°C overnight. Coverslips were then washed three times with PBS and incubated with secondary antibodies at room temperature for 2 h. Cells were finally incubated with 200 nM BODIPY (Sigma) in blocking buffer for 30 min before mounting.

For immunostaining of tissue sections, mice were first given a lethal dose of anesthesia and perfused with PBS followed by 4% PFA. Retinas, optic nerves or DRGs were dissected and postfixed in 4% PFA for 2 h. Tissue was cryoprotected in 30% sucrose overnight and then embedded into OCT compound (Tissue-Tek) at −80°C. Samples were sectioned at −20°C (20 μm for retina and 8 μm for nerve and DRG). Tissue sections were then blocked and permeabilized with 0.1% Triton X-100 in 4% NGS. After the samples were blocked, they were incubated in primary antibody diluted by blocking buffer overnight. After the samples were washed 3 times with PBS, the corresponding secondary antibody diluted by blocking buffer was applied. After the samples were mounted on coverslips, they were imaged under a confocal or epifluorescence (Nikon, TE2000) microscope.

#### Lipid extraction and UPLC-MS

Lipid extraction was performed using the Folch method. Cortical neurons were lysed in a 2:1 chloroform:methanol mixture. After the upper phase was siphoned, the solvent was dried by line blowing with nitrogen. Lipid extracts were analyzed using a Synapt G2 HDMS mass spectrometer coupled with an ACQUITY UPLC system (Waters, Milford, USA). The UPLC separation was carried out using a Charge Surface Hybrid column (particle size: 1.7 μm; length: 100 mm; i.d.: 2.1 mm). The mobile phase consisted of solvent A (0.1% formic acid in water, v/v) and solvent B (0.1% formic acid in acetonitrile, v/v), each with 10 mM ammonium acetate. The elution gradient conditions were as follows: 0 min, 40% B; 2 min, 43% B; 2-2.1 min, 50% B; 12 min, 54% B; 12.1 min, 70% B; 12.1-18 min, 99% B. The flow rate was 0.2 mL min^-1^, and the injection volume was 2 μL. A two minutes post-run time was set to fully equilibrate the column. Column temperature and sample chamber temperature were set to 55°C and 6°C, respectively. The source parameters were set as follows: source temperature, 90°C; desolvation temperature, 400°C; core gas flow, 20 L h^-1^; cone voltage, 40 V; capillary voltage, 3 kV and 2.5 kV in positive and negative ion modes, respectively. The mass range was set as 50 – 1200 Da. The collision energy was set as 40 V. Individual lipid species were semiquantified by referencing to spiked internal standards obtained from Avanti Polar Lipids (Alabaster, AL), i.e., PC (16:0-d31/18:1) and TG (16:0/18:0/16:0-d5).

Raw UPLC-ESI-MS data were directly imported to the Progenesis QI software (Waters-Nonlinear) for data processing, including peak picking, alignment (retention time correction), and data normalization. The processed data matrices were imported to the IBM SPSS Statistics software (Version 11.0, SPSS Inc., Chicago, IL, USA) for t test analysis. The *m/z* values of ions with p value < 0.05 were further exported to the SIMCA 14.1 software for OPLS-DA. From the OPLS-DA, ions with VIP ≥ 1 and fold change between different groups > 1.5 were considered as potential biomarkers and were subjected to identification using a database (LIPIDMAPS, Metlin, LipidBlast and HMDB) and MS/MS fragmentation.

### Quantification and Statistical Analysis

The number of animals or repeats is described in figure legends. All analyses were conducted using Prism 6 software (GraphPad Software, La Jolla, CA). Student’s t test was used for two-group comparisons, and ANOVA was used for multi-group comparisons. An estimate of variation in each group is indicated by the standard error of mean (SEM). ^∗∗^ p ≤ 0.01, ^∗^ p ≤ 0.05.

### Data and Code Availability

This study did not generate any code. The data that support the findings of this study are available from the Lead Contact upon request.

## Supplemental Information

Document S1. Figures S1–S7 and Table S1–S5

Document S2. Article plus Supplemental Information

## References

[bib1] Aldskogius H. (1978). Lipid accumulation in axotomized adult rabbit vagal neurons. Electron microscopical observations. Brain Res..

[bib2] Bazinet R.P., Layé S. (2014). Polyunsaturated fatty acids and their metabolites in brain function and disease. Nat. Rev. Neurosci..

[bib3] Belin S., Nawabi H., Wang C., Tang S., Latremoliere A., Warren P., Schorle H., Uncu C., Woolf C.J., He Z., Steen J.A. (2015). Injury-induced decline of intrinsic regenerative ability revealed by quantitative proteomics. Neuron.

[bib4] Blanquie O., Bradke F. (2018). Cytoskeleton dynamics in axon regeneration. Curr. Opin. Neurobiol..

[bib5] Bloch K. (1965). The biological synthesis of cholesterol. Science.

[bib6] Bloch K. (1992). Sterol molecule: structure, biosynthesis, and function. Steroids.

[bib7] Bradke F., Fawcett J.W., Spira M.E. (2012). Assembly of a new growth cone after axotomy: the precursor to axon regeneration. Nat. Rev. Neurosci..

[bib8] Bylesjo M., Rantalainen M., Cloarec O., Nicholson J.K., Holmes E., Trygg J. (2006). OPLS discriminant analysis: combining the strengths of PLS-DA and SIMCA classification. J. Chemometr..

[bib9] Byrne A.B., Walradt T., Gardner K.E., Hubbert A., Reinke V., Hammarlund M. (2014). Insulin/IGF1 signaling inhibits age-dependent axon regeneration. Neuron.

[bib10] Cartoni R., Norsworthy M.W., Bei F., Wang C., Li S., Zhang Y., Gabel C.V., Schwarz T.L., He Z. (2016). The Mammalian-Specific Protein Armcx1 Regulates Mitochondrial Transport during Axon Regeneration. Neuron.

[bib11] Chandran V., Coppola G., Nawabi H., Omura T., Versano R., Huebner E.A., Zhang A., Costigan M., Yekkirala A., Barrett L. (2016). A Systems-Level Analysis of the Peripheral Nerve Intrinsic Axonal Growth Program. Neuron.

[bib12] Chen H.C., Farese R.V. (2005). Inhibition of triglyceride synthesis as a treatment strategy for obesity: lessons from DGAT1-deficient mice. Arterioscler. Thromb. Vasc. Biol..

[bib13] Chen W., Lu N., Ding Y., Wang Y., Chan L.T., Wang X., Gao X., Jiang S., Liu K. (2017). Rapamycin-Resistant mTOR Activity Is Required for Sensory Axon Regeneration Induced by a Conditioning Lesion. eNeuro.

[bib14] Cho Y., Sloutsky R., Naegle K.M., Cavalli V. (2013). Injury-induced HDAC5 nuclear export is essential for axon regeneration. Cell.

[bib15] Cho Y., Shin J.E., Ewan E.E., Oh Y.M., Pita-Thomas W., Cavalli V. (2015). Activating Injury-Responsive Genes with Hypoxia Enhances Axon Regeneration through Neuronal HIF-1α. Neuron.

[bib16] Craddock C.P., Adams N., Bryant F.M., Kurup S., Eastmond P.J. (2015). PHOSPHATIDIC ACID PHOSPHOHYDROLASE Regulates Phosphatidylcholine Biosynthesis in Arabidopsis by Phosphatidic Acid-Mediated Activation of CTP:PHOSPHOCHOLINE CYTIDYLYLTRANSFERASE Activity. Plant Cell.

[bib17] Csaki L.S., Dwyer J.R., Li X., Nguyen M.H., Dewald J., Brindley D.N., Lusis A.J., Yoshinaga Y., de Jong P., Fong L. (2013). Lipin-1 and lipin-3 together determine adiposity in vivo. Mol. Metab..

[bib18] Curcio M., Bradke F. (2018). Axon Regeneration in the Central Nervous System: Facing the Challenges from the Inside. Annu. Rev. Cell Dev. Biol..

[bib19] DeVita R.J., Pinto S. (2013). Current status of the research and development of diacylglycerol O-acyltransferase 1 (DGAT1) inhibitors. J. Med. Chem..

[bib20] Duan X., Qiao M., Bei F., Kim I.J., He Z., Sanes J.R. (2015). Subtype-specific regeneration of retinal ganglion cells following axotomy: effects of osteopontin and mTOR signaling. Neuron.

[bib21] Etschmaier K., Becker T., Eichmann T.O., Schweinzer C., Scholler M., Tam-Amersdorfer C., Poeckl M., Schuligoi R., Kober A., Chirackal Manavalan A.P. (2011). Adipose triglyceride lipase affects triacylglycerol metabolism at brain barriers. J. Neurochem..

[bib22] Fawcett J.W., Verhaagen J. (2018). Intrinsic Determinants of Axon Regeneration. Dev. Neurobiol..

[bib23] Finck B.N., Gropler M.C., Chen Z., Leone T.C., Croce M.A., Harris T.E., Lawrence J.C., Kelly D.P. (2006). Lipin 1 is an inducible amplifier of the hepatic PGC-1alpha/PPARalpha regulatory pathway. Cell Metab..

[bib24] Gaub P., Tedeschi A., Puttagunta R., Nguyen T., Schmandke A., Di Giovanni S. (2010). HDAC inhibition promotes neuronal outgrowth and counteracts growth cone collapse through CBP/p300 and P/CAF-dependent p53 acetylation. Cell Death Differ..

[bib25] Gibellini F., Smith T.K. (2010). The Kennedy pathway--De novo synthesis of phosphatidylethanolamine and phosphatidylcholine. IUBMB Life.

[bib26] Goldberg J.L. (2003). How does an axon grow?. Genes Dev..

[bib27] Goldberg J.L., Klassen M.P., Hua Y., Barres B.A. (2002). Amacrine-signaled loss of intrinsic axon growth ability by retinal ganglion cells. Science.

[bib28] González C., Cánovas J., Fresno J., Couve E., Court F.A., Couve A. (2016). Axons provide the secretory machinery for trafficking of voltage-gated sodium channels in peripheral nerve. Proc. Natl. Acad. Sci. USA.

[bib29] Han G.S., Wu W.I., Carman G.M. (2006). The Saccharomyces cerevisiae Lipin homolog is a Mg2+-dependent phosphatidate phosphatase enzyme. J. Biol. Chem..

[bib30] Han G.S., Siniossoglou S., Carman G.M. (2007). The cellular functions of the yeast lipin homolog PAH1p are dependent on its phosphatidate phosphatase activity. J. Biol. Chem..

[bib31] Harris T.E., Finck B.N. (2011). Dual function lipin proteins and glycerolipid metabolism. Trends Endocrinol. Metab..

[bib32] He Z., Jin Y. (2016). Intrinsic Control of Axon Regeneration. Neuron.

[bib33] Hellal F., Hurtado A., Ruschel J., Flynn K.C., Laskowski C.J., Umlauf M., Kapitein L.C., Strikis D., Lemmon V., Bixby J. (2011). Microtubule stabilization reduces scarring and causes axon regeneration after spinal cord injury. Science.

[bib34] Hu Y., Park K.K., Yang L., Wei X., Yang Q., Cho K.S., Thielen P., Lee A.H., Cartoni R., Glimcher L.H. (2012). Differential effects of unfolded protein response pathways on axon injury-induced death of retinal ganglion cells. Neuron.

[bib35] Husemann J., Loike J.D., Anankov R., Febbraio M., Silverstein S.C. (2002). Scavenger receptors in neurobiology and neuropathology: their role on microglia and other cells of the nervous system. Glia.

[bib36] Inloes J.M., Hsu K.L., Dix M.M., Viader A., Masuda K., Takei T., Wood M.R., Cravatt B.F. (2014). The hereditary spastic paraplegia-related enzyme DDHD2 is a principal brain triglyceride lipase. Proc. Natl. Acad. Sci. USA.

[bib37] Leon S., Yin Y., Nguyen J., Irwin N., Benowitz L.I. (2000). Lens injury stimulates axon regeneration in the mature rat optic nerve. J. Neurosci..

[bib38] Li S., Yang C., Zhang L., Gao X., Wang X., Liu W., Wang Y., Jiang S., Wong Y.H., Zhang Y., Liu K. (2016). Promoting axon regeneration in the adult CNS by modulation of the melanopsin/GPCR signaling. Proc. Natl. Acad. Sci. USA.

[bib39] Liu K., Tedeschi A., Park K.K., He Z. (2011). Neuronal intrinsic mechanisms of axon regeneration. Annu. Rev. Neurosci..

[bib40] Luo X., Ribeiro M., Bray E.R., Lee D.H., Yungher B.J., Mehta S.T., Thakor K.A., Diaz F., Lee J.K., Moraes C.T. (2016). Enhanced Transcriptional Activity and Mitochondrial Localization of STAT3 Co-induce Axon Regrowth in the Adult Central Nervous System. Cell Rep..

[bib41] Mahar M., Cavalli V. (2018). Intrinsic mechanisms of neuronal axon regeneration. Nat. Rev. Neurosci..

[bib42] Mao C.A., Li H., Zhang Z., Kiyama T., Panda S., Hattar S., Ribelayga C.P., Mills S.L., Wang S.W. (2014). T-box transcription regulator Tbr2 is essential for the formation and maintenance of Opn4/melanopsin-expressing intrinsically photosensitive retinal ganglion cells. J. Neurosci..

[bib43] Meltzer S., Bagley J.A., Perez G.L., O’Brien C.E., DeVault L., Guo Y., Jan L.Y., Jan Y.N. (2017). Phospholipid Homeostasis Regulates Dendrite Morphogenesis in Drosophila Sensory Neurons. Cell Rep..

[bib44] Moore D.L., Blackmore M.G., Hu Y., Kaestner K.H., Bixby J.L., Lemmon V.P., Goldberg J.L. (2009). KLF family members regulate intrinsic axon regeneration ability. Science.

[bib45] Nawabi H., Belin S., Cartoni R., Williams P.R., Wang C., Latremolière A., Wang X., Zhu J., Taub D.G., Fu X. (2015). Doublecortin-Like Kinases Promote Neuronal Survival and Induce Growth Cone Reformation via Distinct Mechanisms. Neuron.

[bib46] Oh Y.M., Mahar M., Ewan E.E., Leahy K.M., Zhao G., Cavalli V. (2018). Epigenetic regulator UHRF1 inactivates REST and growth suppressor gene expression via DNA methylation to promote axon regeneration. Proc. Natl. Acad. Sci. USA.

[bib47] Park K.K., Liu K., Hu Y., Smith P.D., Wang C., Cai B., Xu B., Connolly L., Kramvis I., Sahin M., He Z. (2008). Promoting axon regeneration in the adult CNS by modulation of the PTEN/mTOR pathway. Science.

[bib48] Pascual F., Carman G.M. (2013). Phosphatidate phosphatase, a key regulator of lipid homeostasis. Biochim. Biophys. Acta.

[bib49] Pernet V., Joly S., Dalkara D., Jordi N., Schwarz O., Christ F., Schaffer D.V., Flannery J.G., Schwab M.E. (2013). Long-distance axonal regeneration induced by CNTF gene transfer is impaired by axonal misguidance in the injured adult optic nerve. Neurobiol. Dis..

[bib50] Pfenninger K.H. (2009). Plasma membrane expansion: a neuron’s Herculean task. Nat. Rev. Neurosci..

[bib51] Pfrieger F.W., Ungerer N. (2011). Cholesterol metabolism in neurons and astrocytes. Prog. Lipid Res..

[bib52] Picelli S., Faridani O.R., Björklund A.K., Winberg G., Sagasser S., Sandberg R. (2014). Full-length RNA-seq from single cells using Smart-seq2. Nat. Protoc..

[bib53] Platt R.J., Chen S., Zhou Y., Yim M.J., Swiech L., Kempton H.R., Dahlman J.E., Parnas O., Eisenhaure T.M., Jovanovic M. (2014). CRISPR-Cas9 knockin mice for genome editing and cancer modeling. Cell.

[bib54] Reue K., Zhang P. (2008). The lipin protein family: dual roles in lipid biosynthesis and gene expression. FEBS Lett..

[bib55] Rivieccio M.A., Brochier C., Willis D.E., Walker B.A., D’Annibale M.A., McLaughlin K., Siddiq A., Kozikowski A.P., Jaffrey S.R., Twiss J.L. (2009). HDAC6 is a target for protection and regeneration following injury in the nervous system. Proc. Natl. Acad. Sci. USA.

[bib56] Ryu D., Seo W.Y., Yoon Y.S., Kim Y.N., Kim S.S., Kim H.J., Park T.S., Choi C.S., Koo S.H. (2011). Endoplasmic reticulum stress promotes LIPIN2-dependent hepatic insulin resistance. Diabetes.

[bib57] Santos-Rosa H., Leung J., Grimsey N., Peak-Chew S., Siniossoglou S. (2005). The yeast lipin Smp2 couples phospholipid biosynthesis to nuclear membrane growth. EMBO J..

[bib58] Schmidt T.M., Taniguchi K., Kofuji P. (2008). Intrinsic and extrinsic light responses in melanopsin-expressing ganglion cells during mouse development. J. Neurophysiol..

[bib59] Schönfeld P., Reiser G. (2013). Why does brain metabolism not favor burning of fatty acids to provide energy? Reflections on disadvantages of the use of free fatty acids as fuel for brain. J. Cereb. Blood Flow Metab..

[bib60] Shin J.E., Geisler S., DiAntonio A. (2014). Dynamic regulation of SCG10 in regenerating axons after injury. Exp. Neurol..

[bib61] Siniossoglou S. (2013). Phospholipid metabolism and nuclear function: roles of the lipin family of phosphatidic acid phosphatases. Biochim. Biophys. Acta.

[bib62] Smith S.J., Cases S., Jensen D.R., Chen H.C., Sande E., Tow B., Sanan D.A., Raber J., Eckel R.H., Farese R.V. (2000). Obesity resistance and multiple mechanisms of triglyceride synthesis in mice lacking Dgat. Nat. Genet..

[bib63] Stone S.J., Myers H.M., Watkins S.M., Brown B.E., Feingold K.R., Elias P.M., Farese R.V. (2004). Lipopenia and skin barrier abnormalities in DGAT2-deficient mice. J. Biol. Chem..

[bib64] Strakova J., Demizieux L., Campenot R.B., Vance D.E., Vance J.E. (2011). Involvement of CTP:phosphocholine cytidylyltransferase-β2 in axonal phosphatidylcholine synthesis and branching of neurons. Biochim. Biophys. Acta.

[bib65] Sun F., Park K.K., Belin S., Wang D., Lu T., Chen G., Zhang K., Yeung C., Feng G., Yankner B.A., He Z. (2011). Sustained axon regeneration induced by co-deletion of PTEN and SOCS3. Nature.

[bib66] Sweeney N.T., Tierney H., Feldheim D.A. (2014). Tbr2 is required to generate a neural circuit mediating the pupillary light reflex. J. Neurosci..

[bib67] Swiech L., Heidenreich M., Banerjee A., Habib N., Li Y., Trombetta J., Sur M., Zhang F. (2015). In vivo interrogation of gene function in the mammalian brain using CRISPR-Cas9. Nat. Biotechnol..

[bib68] Terenzio M., Koley S., Samra N., Rishal I., Zhao Q., Sahoo P.K., Urisman A., Marvaldi L., Oses-Prieto J.A., Forester C. (2018). Locally translated mTOR controls axonal local translation in nerve injury. Science.

[bib69] Tozawa R., Ishibashi S., Osuga J., Yagyu H., Oka T., Chen Z., Ohashi K., Perrey S., Shionoiri F., Yahagi N. (1999). Embryonic lethality and defective neural tube closure in mice lacking squalene synthase. J. Biol. Chem..

[bib70] Vance J.E., Campenot R.B., Vance D.E. (2000). The synthesis and transport of lipids for axonal growth and nerve regeneration. Biochim. Biophys. Acta.

[bib71] Wakil S.J. (1989). Fatty acid synthase, a proficient multifunctional enzyme. Biochemistry.

[bib72] Weiss S.B., Kennedy E.P., Kiyasu J.Y. (1960). The enzymatic synthesis of triglycerides. J. Biol. Chem..

[bib73] Welte M.A. (2015). Expanding roles for lipid droplets. Curr. Biol..

[bib74] Weng Y.L., An R., Cassin J., Joseph J., Mi R., Wang C., Zhong C., Jin S.G., Pfeifer G.P., Bellacosa A. (2017). An Intrinsic Epigenetic Barrier for Functional Axon Regeneration. Neuron.

[bib75] Weng Y.L., Wang X., An R., Cassin J., Vissers C., Liu Y., Liu Y., Xu T., Wang X., Wong S.Z.H. (2018). Epitranscriptomic m(6)A Regulation of Axon Regeneration in the Adult Mammalian Nervous System. Neuron.

[bib76] Wu Y., Whiteus C., Xu C.S., Hayworth K.J., Weinberg R.J., Hess H.F., De Camilli P. (2017). Contacts between the endoplasmic reticulum and other membranes in neurons. Proc. Natl. Acad. Sci. USA.

[bib77] Yen C.L., Stone S.J., Koliwad S., Harris C., Farese R.V. (2008). Thematic review series: glycerolipids. DGAT enzymes and triacylglycerol biosynthesis. J. Lipid Res..

[bib78] Zhang P., Reue K. (2017). Lipin proteins and glycerolipid metabolism: Roles at the ER membrane and beyond. Biochim Biophys Acta Biomembr.

[bib79] Zhang P., Takeuchi K., Csaki L.S., Reue K. (2012). Lipin-1 phosphatidic phosphatase activity modulates phosphatidate levels to promote peroxisome proliferator-activated receptor γ (PPARγ) gene expression during adipogenesis. J. Biol. Chem..

[bib80] Zhang P., Verity M.A., Reue K. (2014). Lipin-1 regulates autophagy clearance and intersects with statin drug effects in skeletal muscle. Cell Metab..

[bib81] Zhang P., Csaki L.S., Ronquillo E., Baufeld L.J., Lin J.Y., Gutierrez A., Dwyer J.R., Brindley D.N., Fong L.G., Tontonoz P. (2019). Lipin 2/3 phosphatidic acid phosphatases maintain phospholipid homeostasis to regulate chylomicron synthesis. J. Clin. Invest..

[bib82] Zhou B., Yu P., Lin M.Y., Sun T., Chen Y., Sheng Z.H. (2016). Facilitation of axon regeneration by enhancing mitochondrial transport and rescuing energy deficits. J. Cell Biol..

[bib83] Ziegler A.B., Thiele C., Tenedini F., Richard M., Leyendecker P., Hoermann A., Soba P., Tavosanis G. (2017). Cell-Autonomous Control of Neuronal Dendrite Expansion via the Fatty Acid Synthesis Regulator SREBP. Cell Rep..

