# Supplementary material for: Rewiring Neuronal Glycerolipid Metabolism Determines the Extent of Axon Regeneration
Source: Neuron. 2020 Jan 22;105(2):276–292.e5. doi: 10.1016/j.neuron.2019.10.009 (PMC6975164; doi:10.1016/j.neuron.2019.10.009)
Supplement: Document S1. Figures S1–S7 and Table S1–S5 [file mmc1.pdf]

Neuron, Volume *105*

Supplemental Information

Rewiring Neuronal Glycerolipid Metabolism

Determines the Extent of Axon Regeneration

Chao Yang, Xu Wang, Jianying Wang, Xuejie Wang, Weitao Chen, Na Lu, Symeon Siniossoglou, Zhongping Yao, and Kai Liu

SUPPLEMENTAL INFORMATION

Rewiring neuronal glycerolipid metabolism determines the extent of axon regeneration

Chao Yang, Xu Wang, Jianying Wang, Xuejie Wang, Weitao Chen, Na Lu, Symeon Siniossoglou, Zhongping Yao, Kai Liu

Supplemental Figures 1-7

Supplemental Tables 1-5

Figure S1. In vitro screening and in vivo validation of lipin1 as an intrinsic suppressor of axon regeneration, Related to Figure 1.

(A) Time course of the in-vivo optic nerve injury model and the in-vitro DRG replating model. (B) Quantification of the axon elongation by in vitro screening of cholesterol and fatty acid metabolic genes in adult DRG neurons. We tested 6 genes including *Hmgcr1, Hmgcs, Fdft1, CD36, Acc1, Acc2*. Hmgcr1: 3-hydroxy-3-methylglutaryl-coenzyme A reductase-1. Hmgcs: 3-Hydroxy-3-Methylglutaryl-CoA Synthase. Fdft1: Farnesyl-Diphosphate Farnesyltransferase 1. CD36: Fatty acid translocase. Acc: Acetyl-CoA carboxylase. Adult DRG neurons were dissociated and transfected with the plasmids for three days. Neurons were then replated and fixed 24 h after replating. DRG neurites were visualized by Tuj1 staining. Three mice and 10-20 neurons from each mouse were quantified in each group. ANOVA followed by Dunnett's test. (C) Retinal sections from control and lipin1 KD mice stained with Tuj1 antibody (green) and lipin1 antibody (red). Scale bar: 10 μm. (D) Whole-mount retinas from control and lipin1 KD mice at 2 WPI with Tuj1 (green) staining to show the surviving RGCs. Scale bar: 50 μm. (E) Quantification of RGC survival in (D). Student's t-test. ns, not significant. (F) Gel images of T7E1-treated PCR products amplified from the target sites of lipin1 in Neuro2A cells transfected by SpCas9 and corresponding sgRNA. (G) Retinal sections from Rosa26-Cas9 mice injected with either AAV-control-sgRNA or AAV-lipin1-sgRNA and stained for Tuj1 (green) and lipin1 (red). Scale bar: 50 μm. Arrows indicate lipin1 negative RGCs. (H) Quantification of lipin1 negative RGCs from Rosa26-Cas9 mice injected with either AAV-control-sgRNA or AAV-lipin1-sgRNA. ** $P \leq 0.01$, Student's t-test. (I) Quantification of surviving RGCs from Rosa26-Cas9 mice injected with either AAV-control-sgRNA or AAV-lipin1-sgRNA. ns, not significant, Student's t-test. (J) Quantification of expression of lipin2 in sorted RGCs detected by qRT-PCR. Each sample was run in quadruplicate in each assay. GAPDH was used as the endogenous control. ns, not significant, Student's t-test. (K) Sections of optic nerves from Rosa26-Cas9 mice at 2 WPI injected with respective AAVs. Scale bar: 100 μm. (L) Number of regenerating axons at the indicated distances distal to the lesion site. ** $P \leq 0.01$, ANOVA followed by Tukey's test, n= 5 mice. (M) Sections of optic

nerves from pten floxed mice at 2 WPI injected with AAV-Cre combined with either AAV-control or lipin1-shRNA. Scale bar: 100 μm. (N) Number of regenerating axons at indicated distances distal to the lesion site. * $P \leq 0.05$, ANOVA followed by Tukey's test, n= 6 mice. (O) Validation of the KD efficiency of two lipin1 shRNA by doing western blot in Neuro2A cells after transfection. GAPDH was used as the loading control. Three batches of experiments were used for quantification. ** $P \leq 0.01$, * $P \leq 0.05$, ANOVA followed by Tukey's test. (P) Number of regenerating axons from WT mice at 2 WPI injected with AAV-control or lipin1-shRNA at indicated distances from the lesion site. ** $P \leq 0.01$, * $P \leq 0.05$, ANOVA followed by Tukey's test, n= 6 mice. Error bars indicate SEM.

Figure S2. Selective regulation of lipin1 levels in different types of RGCs after optic nerve injury, Related to Figure 2.

(A) Whole-mount retinas from *Opn4*-GFP mice three days after axotomy or sham surgery were collected and stained for DAPI (blue) and lipin1 (red). GFP (green) labeled M1-M3 ipRGCs. Scale bar: 50 μm. (B) Percentage of M1-M3 ipRGCs with a low or high lipin1 level indicated by lipin1 staining. ANOVA followed by Bonferroni's test. (C) Whole-mount retinas from WT mice three days after axotomy or sham surgery were collected and stained for DAPI (blue), TBR2 (green) and lipin1 (red). Scale bar: 50 μm. (D) Percentage of TBR2+ RGCs with a low or high lipin1 level indicated by lipin1 staining. ANOVA followed by Bonferroni's test. (E) A whole-mount retina from a mouse with AAV-lipin1-shRNA injection showing regenerating RGCs retrogradely labeled with FG 2 WPI. The retina was stained for SMI32 (red) and FG (green). Scale bar: 50 μm. (F) Whole-mount retinas with Pten deletion, CNTF overexpression or from control mice with AAV-GFP injection three days after optic nerve crush were collected and stained with SMI32 (green) and lipin1 (red) antibodies. Scale bar: 50 μm. Zoom-in images are shown in the right panel. Scale bar: 10 μm. (G) Percentages of αRGCs with a low or high lipin1 level indicated by lipin1 staining. ** $P \leq 0.01$, ANOVA followed by Bonferroni's test. Error bars indicate SEM.

Figure S3. Lipin1 inhibits axon elongation in vitro through its phosphatidate phosphatase activity and regulates glycerolipid metabolism in neurons, Related to Figure 3.

(A) A whole-mount retina from WT mice injected with AAV-lipin1-shRNA and AAV-lipin1-WT with Tuj1 (gray) and HA-tag (red) staining. AAV- lipin1-shRNA -infected RGCs were labeled by GFP (green) and AAV-lipin1-WT-infected RGCs were labeled by HA-tag (red). Scale bar: 50 μm. (B) Quantification of surviving RGCs from WT mice at 2 WPI, injected with AAV-lipin1-shRNA combined with AAV-GFP, AAV-lipin1-WT, AAV-lipin1-PAPm or AAV-lipin1-ΔNLS. Tuj1 staining was used to label RGCs. ANOVA followed by Dunnett's test. (C) Adult DRG neurons were dissociated and cultured with different AAVs for 10 days. Neurons were then replated and fixed 24 h later. DRG neurites were visualized by Tuj1 staining. Quantification of the length of the longest axon for each DRG neuron in respective groups. ** $P \leq 0.01$, * $P \leq 0.05$, ANOVA followed by Tukey's test. (D) Representative images of cortical neurons with DMSO vehicle or AraC treatment. Neurons were stained for Tuj1 (red) or GFAP (green). Scale bar: 400 μm. (E-H) Levels of individual TG, PC, PE, CE, and FA species normalized to the total protein from either Ctrl or lipin1-shRNA group. The molecular species are indicated as the total number of carbons: the number of double bonds. ** $P \leq 0.01$, * $P \leq 0.05$, t-test. Error bars indicate SEM.

Figure S4. The knockdown efficacy of shRNAs against *Atgl* and *Ddhd2*, and regeneration requires TG hydrolysis, Related to Figure 4.

(A) Validation of *Atgl* shRNA efficiency in Neuro2A cells. Western blots of lysates of N2a cells transfected with control or *Atgl* shRNA for two days are shown. GAPDH was used as the loading control. (B) Quantification of band intensity in (A). Three batches of experiments were used for quantification. * $P \leq 0.05$, Student's t-test. (C) Validation of *Ddhd2* shRNA efficiency in Neuro2A cells. Shown are western blots of lysates of N2a cells transfected with control or *Ddhd*2 shRNA for two days. GAPDH was used as the loading control. (D) Quantification of band intensity in (C). Three batches of experiments were used for quantification. * $P \leq 0.05$, Student's t-test. (E) A whole-mount retina from WT mice injected with AAV-lipin1-sgRNA and AAV-*Atgl*-

shRNA with Tuj1 (gray) staining. AAV- lipin1-sgRNA -infected RGCs were labeled by mCherry (red) and AAV-*Atgl*-shRNA-infected RGCs were labeled by GFP (green). Scale bar: 50 μm. (F) Quantification of surviving RGCs at 2 WPI, injected with the corresponding AAVs. Tuj1 staining was used to label RGCs. ANOVA followed by Dunnett's test. (G) Quantification of surviving RGCs from the respective groups. ANOVA followed by Dunnett's test. (H) Sections of optic nerves from WT mice at 2 WPI injected with AAV-CNTF combined with either AAV-control or *Atgl*-shRNA. Scale bar: 100 μm. (I) Number of regenerating axons at indicated distances from the lesion site. ** $P \leq 0.01$, ANOVA followed by Bonferroni's test, n= 6 mice. (J) Sections of optic nerves from pten floxed mice at 2 WPI injected with AAV-Cre combined with either AAV-control or *Atgl*-shRNA. Scale bar: 100 μm. (K) Number of regenerating axons at indicated distances distal to the lesion site. ** $P \leq 0.01$, ANOVA followed by Bonferroni's test, n= 6 mice.

Figure S5. TG synthesis inhibition promotes axon elongation in vitro, and regeneration requires TG hydrolysis, Related to Figure 5.

(A) Representative images of replated neurons from the respective groups with Tuj1 staining. Adult DRG neurons were dissociated and cultured with the respective drug for five days. Neurons were then replated and fixed 24 h later. DRG neurites were visualized by Tuj1 staining. Scale bar: 400 μm. (B) Quantification of the length of the longest axon for each DRG neuron in (A). Three mice and 10-20 neurons from each mouse were quantified in each group. * $P \leq 0.05$, ANOVA followed by Dunnett's test. (C) Representative images of replated neurons from the respective groups with Tuj1 staining. Adult DRG neurons were dissociated and cultured with different AAV shRNA for 10 days. Neurons were then replated and fixed 24 h later. DRG neurites were visualized by Tuj1 staining. Scale bar: 400 μm. (D) Quantification of the lengths of the longest axon for each DRG neuron in (C). Ten to twenty cells from each mouse and 3 mice were quantified in each group. ** $P \leq 0.01$, * $P \leq 0.05$, ANOVA followed by Dunnett's test. (E) Gel images of T7E1-treated PCR products amplified from the target sites of *Dgat1* or *Dgat2* in Neuro2A cells transfected by SpCas9 and corresponding sgRNA. (F) Quantification of whole-mount retinas from respective groups at 2 WPI. Tuj1 staining to show the number of surviving RGCs. ANOVA

followed by Dunnett's test. (G) Sections of optic nerves from Rosa26-Cas9 mice at 2 WPI, injected with AAV-*Dgat1* or *Dgat2*-sgRNA combined with AAV-control or *Atgl* shRNA. Axons were labeled by CTB-FITC. Scale bar: 100 μm. (H) Quantification of whole-mount retinas from the respective groups at 2 WPI, with Tuj1 staining to show the number of surviving RGCs. ANOVA followed by Dunnett's test. (I) Number of regenerating axons at the indicated distances distal to the lesion site. ** $P \leq 0.01$, * $P \leq 0.05$, ANOVA followed by Tukey's test, n= 5 mice. Error bars indicate SEM.

Figure S6. The effect of knocking down individual PL biosynthesis genes on axon elongation in vitro and axon regeneration induced by Dgat1 depletion; Expression of the glycerolipid pathway enzymes in subtypes of RGCs after injury, Related to Figure 6.

(A) Representative images of replated neurons from the respective groups with Tuj1 staining. Adult DRG neurons were dissociated and cultured with the respective virus for 10 days. Neurons were then replated and fixed 24 h later. DRG neurites were visualized by Tuj1 staining. Scale bar: 400 μm. (B) Quantification of the lengths of the longest axon for each DRG neuron in (A) ANOVA followed by Dunnett's test. (C) Quantification of axon regeneration in injured optic nerves from WT mice injected with the corresponding virus. Shown are numbers of regenerating axons at the indicated distances distal to the lesion site. ANOVA followed by Tukey's test. n= 6 mice. (D) Quantification of whole-mount retinas from the respective groups at 2 WPI, with Tuj1 staining to show the number of surviving RGCs. ANOVA followed by Dunnett's test. (E) Quantification of axon regeneration in injured optic nerves from Rosa26-Cas9 mice injected with the corresponding virus. Shown are numbers of regenerating axons at the indicated distances distal to the lesion site. ** $P \leq 0.01$, ANOVA followed by Tukey's test, n= 6 mice. (F) Quantification of axon regeneration in injured optic nerves from WT mice injected with AAV-CNTF and AAV-shCtrl or AAV-sh*Pcyt1b*. Shown are numbers of regenerating axons at the indicated distances distal to the lesion site. ** $P \leq 0.01$, ANOVA followed by Bonferroni's test, n= 6 mice. (G) Quantification of axon regeneration in injured optic nerves from *Pten*-floxed mice injected with AAV-Cre and AAV-shCtrl or AAV-sh*Pcyt1b*. Shown are numbers of regenerating axons at the indicated distances distal to the lesion site. ** $P \leq 0.01$,

ANOVA followed by Bonferroni's test, n= 3 mice. (H) A whole-mount retina from WT mice injected with AAV-*Pcyt2* with Tuj1 (green) and HA-tag (red) staining. Scale bar: 50 μm. (I) Quantification of expression of different glycerolipid pathway enzymes in αRGCs or M1-M3 ipRGCs detected by qRT-PCR. 12-15 cells were in each group and each cell was run in two replicates. ** $P \leq 0.01$, * $P \leq 0.05$, ANOVA followed by Tukey's test. Error bars indicate SEM.

Figure S7. Lipin1 levels are maintained in DRG neurons after axotomy and the effect of lipin1 KD on the peripheral axon regeneration, Related to Figure 7.
(A) DRG sections from WT animals three days after sciatic nerve crush or sham surgery, stained with Tuj1 (green) or lipin1 (red) antibodies. Scale bar: 100 μm. Zoomed-in images are shown in the lower panel. Scale bar: 20 μm. (B) DRG sections from WT animals 4 weeks after AAV-lipin1 shRNA intraspinal injection. Sections were stained for GFP (green) and Tuj1 (red). Scale bar: 200 μm. (C) Sections of sciatic nerves 3 days after injury from WT animals with AAV-ctrl or lipin1 shRNA intraspinal injection. Axons are visualized by SCG10 staining. Scale bar: 1mm. (D) Quantification of regenerating sensory axons in (B). ns, not significant, Student's t-test. (E) Quantification of the length of the longest axon from the replated neurons with AAV-shCtrl, AAV-shLipin1 or AAV-sh*Ddhd2* treatment. Three mice and 10-20 cells from each mouse were quantified in each group. ** $P \leq 0.01$, ANOVA followed by Dunnett's test. (F) Time course of compound delivery and in-vivo sciatic nerve injury model. (G) DRG sections from WT animals with respective compound injection at two days after sciatic nerve crush, stained with Tuj1 (green) or cleaved caspase3 (red) antibodies. Scale bar: 200 μm.

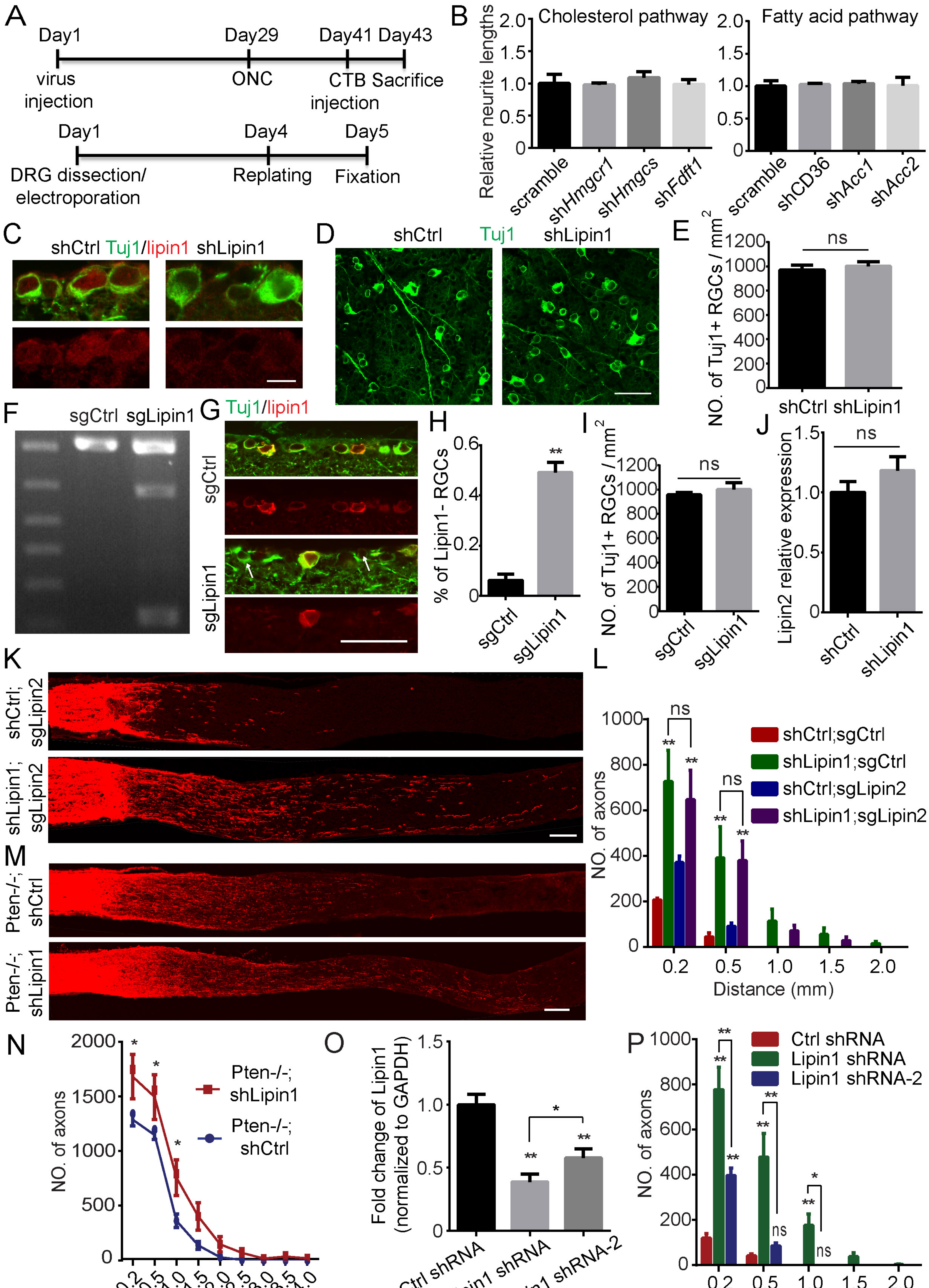

A
Day1
Day29
Day41 Day43
virus injection
ONC
CTB injection
Sacrifice
Day1
Day4
Day5
DRG dissection/ electroporation
Replating
Fixation
B
Relative neurite lengths
Cholesterol pathway
Fatty acid pathway
scramble
shHmgcr1
shHmgcs
shFdft1
scramble
shCD36
shAcc1
shAcc2
C
shCtrl Tuj1/lipin1 shLipin1
D
shCtrl Tuj1 shLipin1
E
NO. of Tuj1+ RGCs / mm2
ns
shCtrl shLipin1
F
sgCtrl sgLipin1
G
Tuj1/lipin1
sgCtrl
sgLipin1
H
% of Lipin1- RGCs
**
sgCtrl
sgLipin1
I
NO. of Tuj1+ RGCs / mm2
ns
sgCtrl
sgLipin1
J
Lipin2 relative expression
ns
shCtrl
shLipin1
K
shCtrl; sgLipin2
shLipin1; sgLipin2
L
NO. of axons
Distance (mm)
shCtrl;sgCtrl
shLipin1;sgCtrl
shCtrl;sgLipin2
shLipin1;sgLipin2
ns
**
M
Pten-/-; shCtrl
Pten-/-; shLipin1
N
NO. of axons
Distance (mm)
Pten-/-; shLipin1
Pten-/-; shCtrl
*
O
Fold change of Lipin1 (normalized to GAPDH)
Ctrl shRNA
Lipin1 shRNA
Lipin1 shRNA-2
*
**
P
NO. of axons
Distance (mm)
Ctrl shRNA
Lipin1 shRNA
Lipin1 shRNA-2
**
*
ns

Figure S1

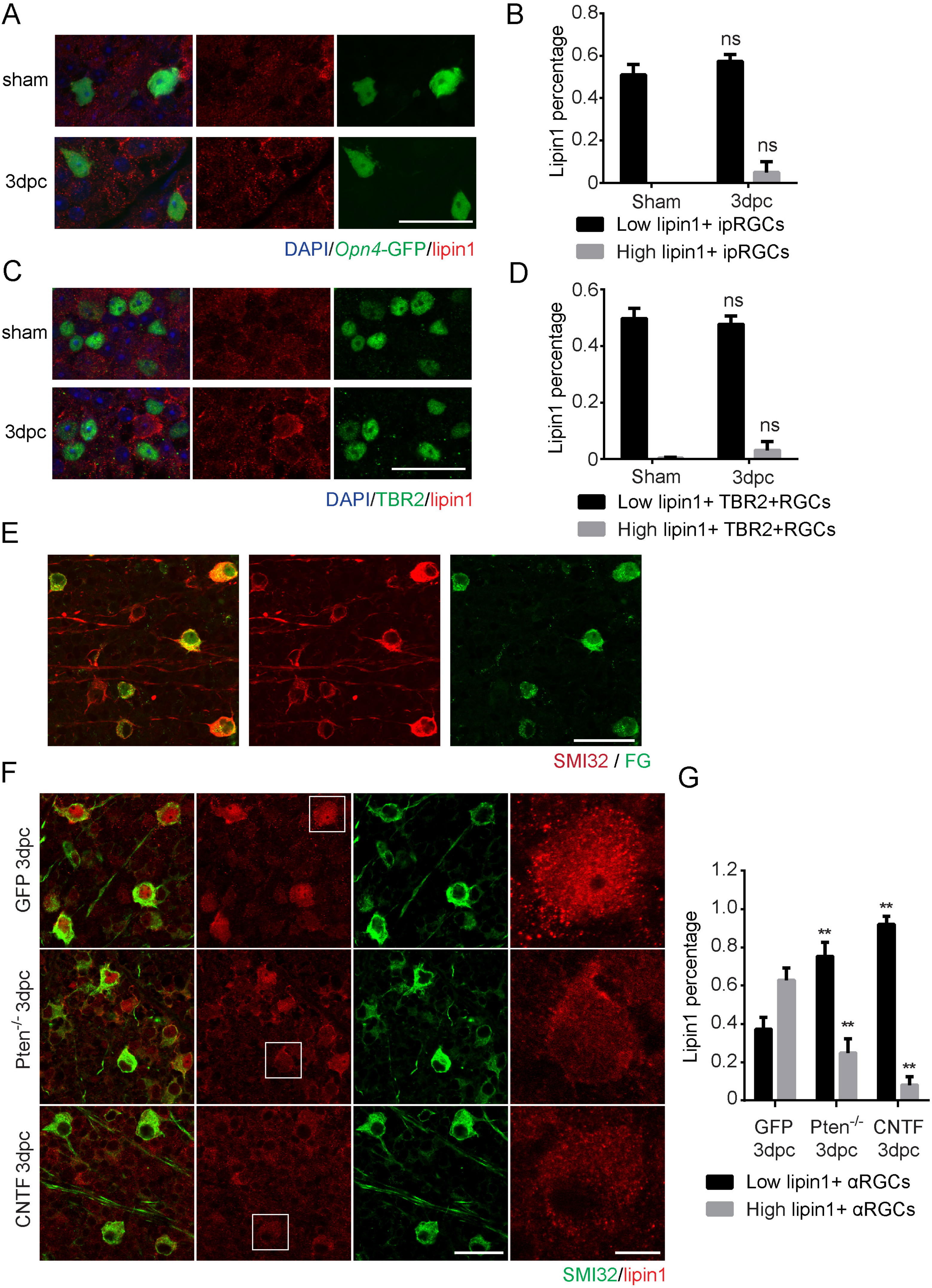

A
sham
3dpc
DAPI/Opn4-GFP/lipin1
B
Lipin1 percentage
0.8
0.6
0.4
0.2
0
ns
ns
Sham
3dpc
Low lipin1+ ipRGCs
High lipin1+ ipRGCs
C
sham
3dpc
DAPI/TBR2/lipin1
D
Lipin1 percentage
0.6
0.4
0.2
0
ns
ns
Sham
3dpc
Low lipin1+ TBR2+RGCs
High lipin1+ TBR2+RGCs
E
SMI32 / FG
F
GFP 3dpc
Pten-/- 3dpc
CNTF 3dpc
SMI32/lipin1
G
Lipin1 percentage
1.2
1.0
0.8
0.6
0.4
0.2
0
**
**
**
**
GFP 3dpc
Pten-/- 3dpc
CNTF 3dpc
Low lipin1+ αRGCs
High lipin1+ αRGCs

Figure S2

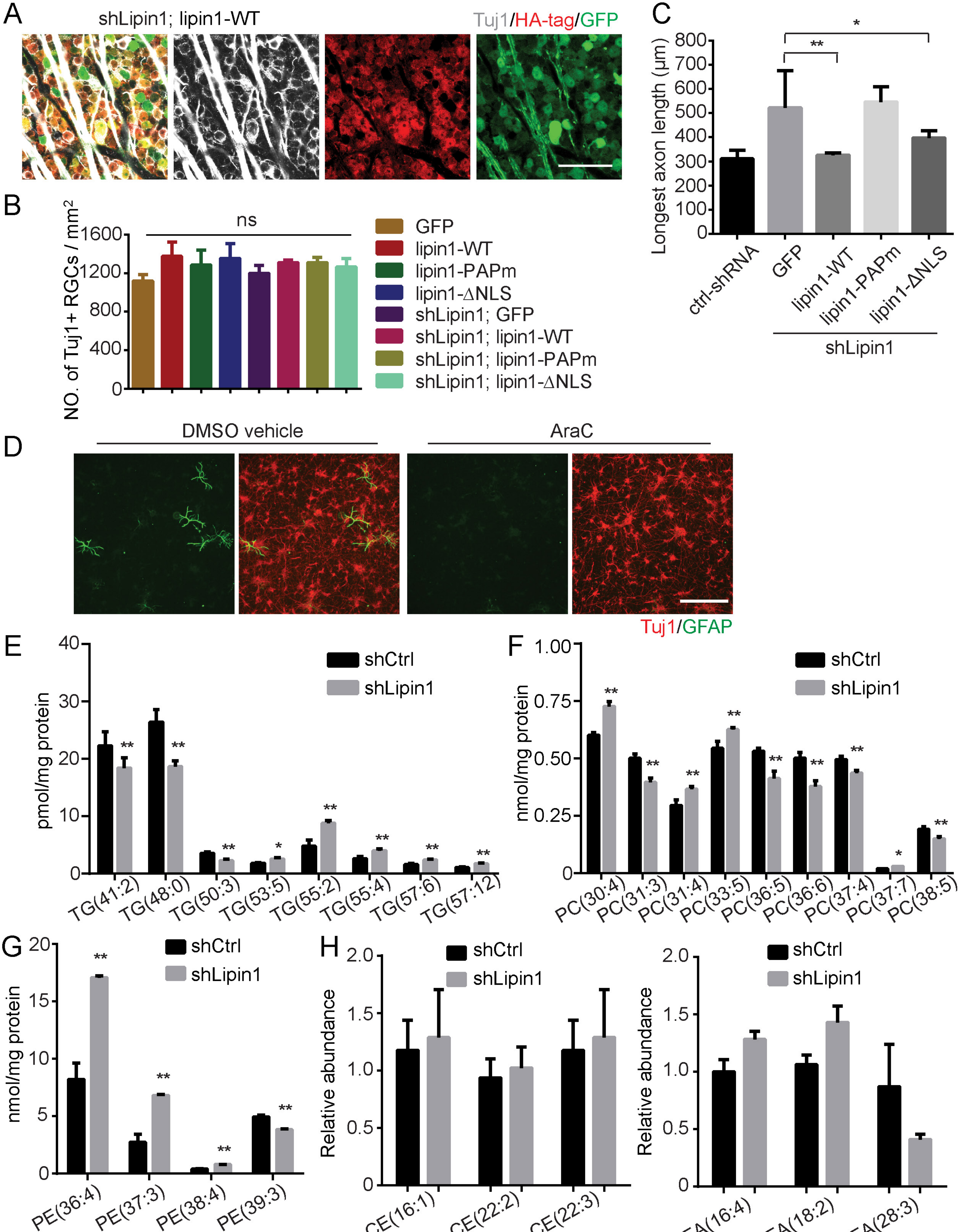
A
shLipin1; lipin1-WT
Tuj1/HA-tag/GFP
B
NO. of Tuj1+ RGCs / mm2
ns
GFP
lipin1-WT
lipin1-PAPm
lipin1-ΔNLS
shLipin1; GFP
shLipin1; lipin1-WT
shLipin1; lipin1-PAPm
shLipin1; lipin1-ΔNLS
C
Longest axon length (μm)
ctrl-shRNA
GFP
lipin1-WT
lipin1-PAPm
lipin1-ΔNLS
shLipin1
D
DMSO vehicle
AraC
Tuj1/GFAP
E
pmol/mg protein
shCtrl
shLipin1
TG(41:2)
TG(48:0)
TG(50:3)
TG(53:5)
TG(55:2)
TG(55:4)
TG(57:6)
TG(57:12)
F
nmol/mg protein
shCtrl
shLipin1
PC(30:4)
PC(31:3)
PC(31:4)
PC(33:5)
PC(36:5)
PC(36:6)
PC(37:4)
PC(37:7)
PC(38:5)
G
nmol/mg protein
shCtrl
shLipin1
PE(36:4)
PE(37:3)
PE(38:4)
PE(39:3)
H
Relative abundance
shCtrl
shLipin1
CE(16:1)
CE(22:2)
CE(22:3)
Relative abundance
shCtrl
shLipin1
FA(16:4)
FA(18:2)
FA(28:3)

Figure S3

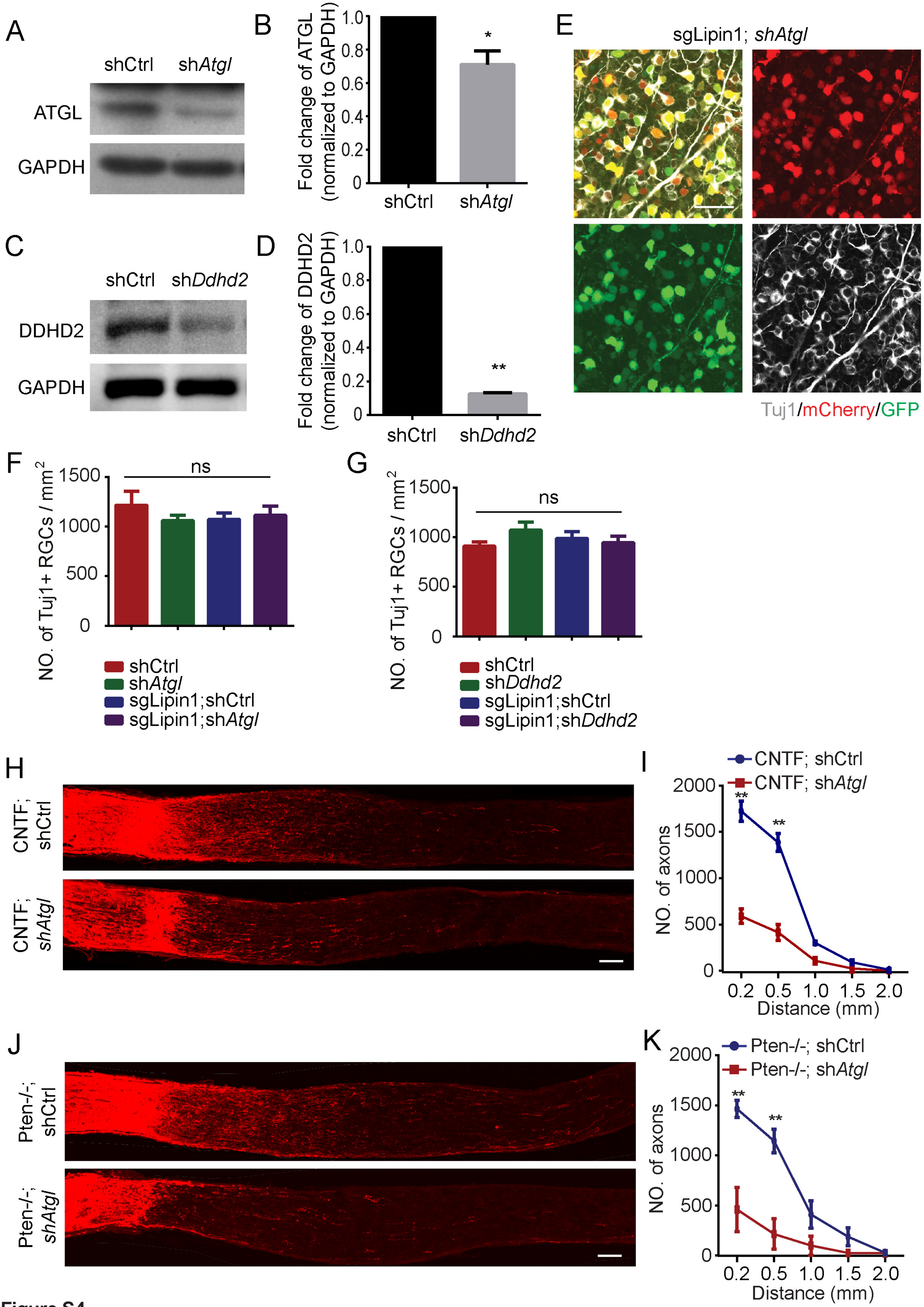

A
shCtrl
shAtgl
ATGL
GAPDH
B
Fold change of ATGL
(normalized to GAPDH)
1.0
0.8
0.6
0.4
0.2
0
*
shCtrl
shAtgl
E
sgLipin1; shAtgl
Tuj1/mCherry/GFP
C
shCtrl
shDdhd2
DDHD2
GAPDH
D
Fold change of DDHD2
(normalized to GAPDH)
**
shCtrl
shDdhd2
F
NO. of Tuj1+ RGCs / mm2
1500
1000
500
0
ns
shCtrl
shAtgl
sgLipin1;shCtrl
sgLipin1;shAtgl
G
NO. of Tuj1+ RGCs / mm2
ns
shCtrl
shDdhd2
sgLipin1;shCtrl
sgLipin1;shDdhd2
H
CNTF; shCtrl
CNTF; shAtgl
I
CNTF; shCtrl
CNTF; shAtgl
2000
1500
1000
500
0
NO. of axons
**
**
0.2 0.5 1.0 1.5 2.0
Distance (mm)
J
Pten-/-; shCtrl
Pten-/-; shAtgl
K
Pten-/-; shCtrl
Pten-/-; shAtgl
NO. of axons
**
**
0.2 0.5 1.0 1.5 2.0
Distance (mm)

Figure S4

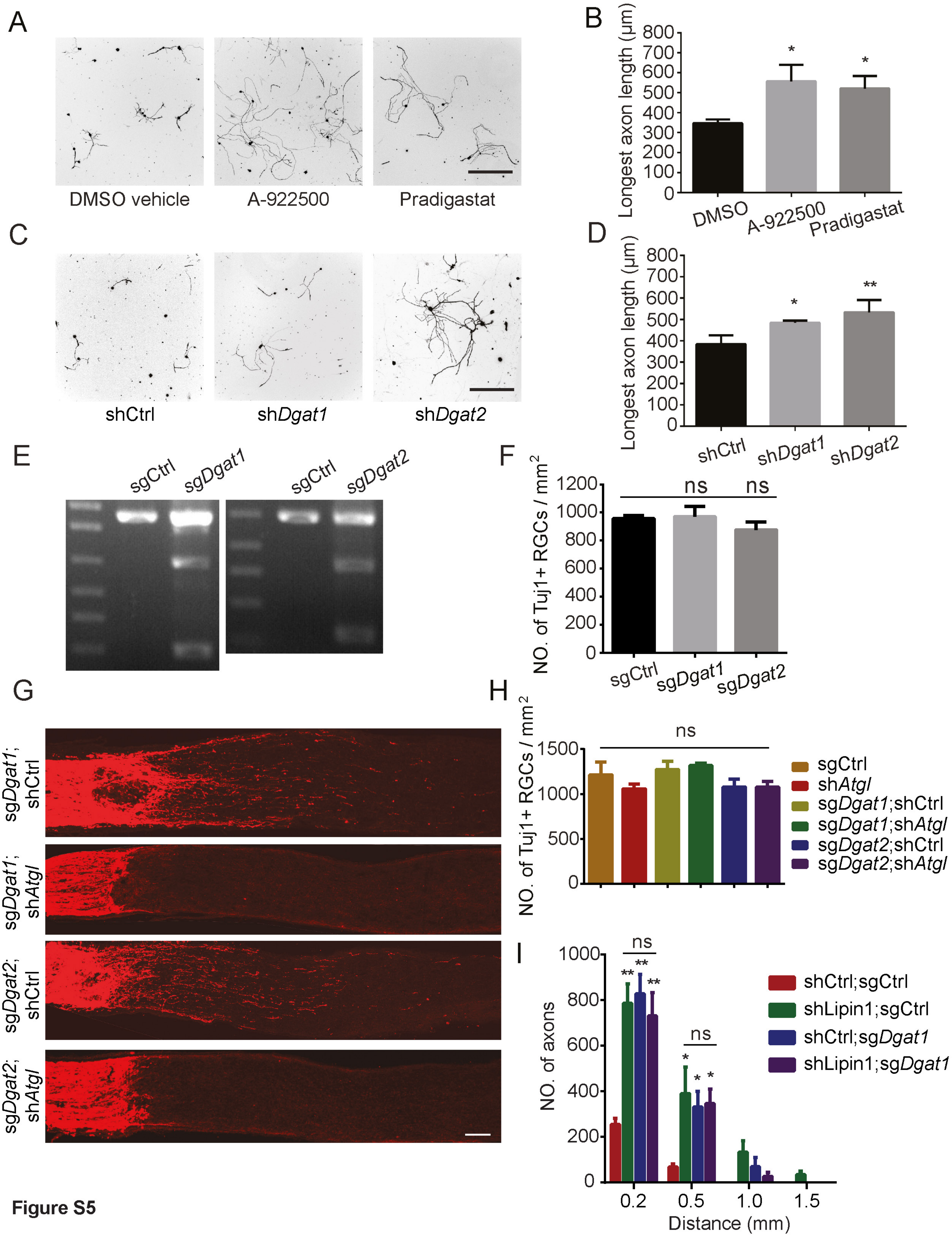
A
DMSO vehicle
A-922500
Pradigastat
B
Longest axon length (μm)
800
700
600
500
400
300
200
100
0
*
*
DMSO
A-922500
Pradigastat
C
shCtrl
shDgat1
shDgat2
D
Longest axon length (μm)
*
**
shCtrl
shDgat1
shDgat2
E
sgCtrl
sgDgat1
sgCtrl
sgDgat2
F
NO. of Tuj1+ RGCs / mm2
1200
1000
800
600
400
200
0
ns
ns
sgCtrl
sgDgat1
sgDgat2
G
sgDgat1; shCtrl
sgDgat1; shAtgl
sgDgat2; shCtrl
sgDgat2; shAtgl
H
NO. of Tuj1+ RGCs / mm2
1500
1000
500
0
ns
sgCtrl
shAtgl
sgDgat1;shCtrl
sgDgat1;shAtgl
sgDgat2;shCtrl
sgDgat2;shAtgl
I
NO. of axons
1000
800
600
400
200
0
ns
**
**
**
ns
*
*
*
shCtrl;sgCtrl
shLipin1;sgCtrl
shCtrl;sgDgat1
shLipin1;sgDgat1
0.2
0.5
1.0
1.5
Distance (mm)

Figure S5

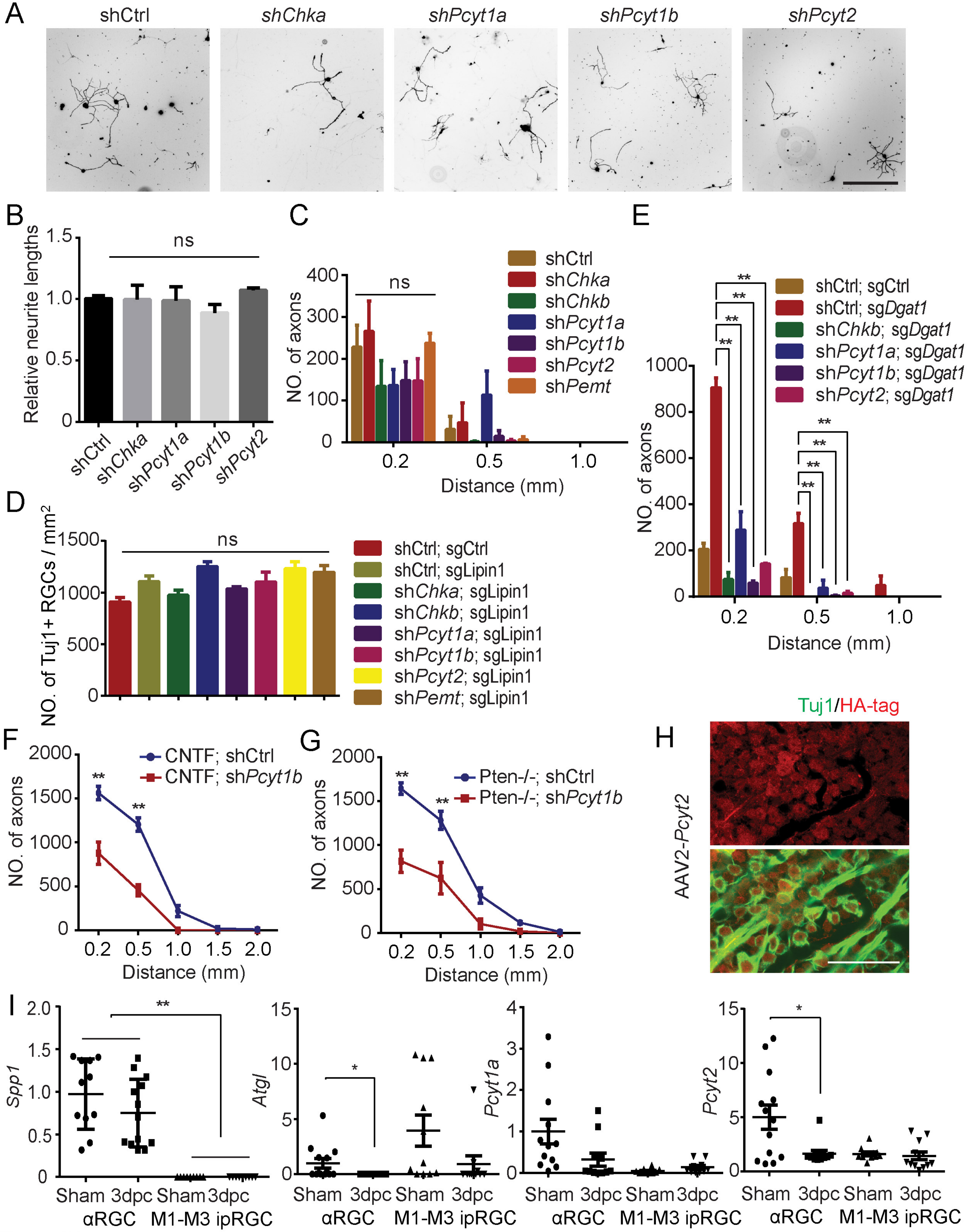
A
shCtrl
shChka
shPcyt1a
shPcyt1b
shPcyt2
B
Relative neurite lengths
ns
shCtrl
shChka
shPcyt1a
shPcyt1b
shPcyt2
C
NO. of axons
ns
Distance (mm)
shCtrl
shChka
shChkb
shPcyt1a
shPcyt1b
shPcyt2
shPemt
E
NO. of axons
Distance (mm)
shCtrl; sgCtrl
shCtrl; sgDgat1
shChkb; sgDgat1
shPcyt1a; sgDgat1
shPcyt1b; sgDgat1
shPcyt2; sgDgat1
D
NO. of Tuj1+ RGCs / mm2
ns
shCtrl; sgCtrl
shCtrl; sgLipin1
shChka; sgLipin1
shChkb; sgLipin1
shPcyt1a; sgLipin1
shPcyt1b; sgLipin1
shPcyt2; sgLipin1
shPemt; sgLipin1
F
NO. of axons
CNTF; shCtrl
CNTF; shPcyt1b
Distance (mm)
G
NO. of axons
Pten-/-; shCtrl
Pten-/-; shPcyt1b
Distance (mm)
H
Tuj1/HA-tag
AAV2-Pcyt2
I
Spp1
Atgl
Pcyt1a
Pcyt2
Sham 3dpc Sham 3dpc
αRGC M1-M3 ipRGC

Figure S6

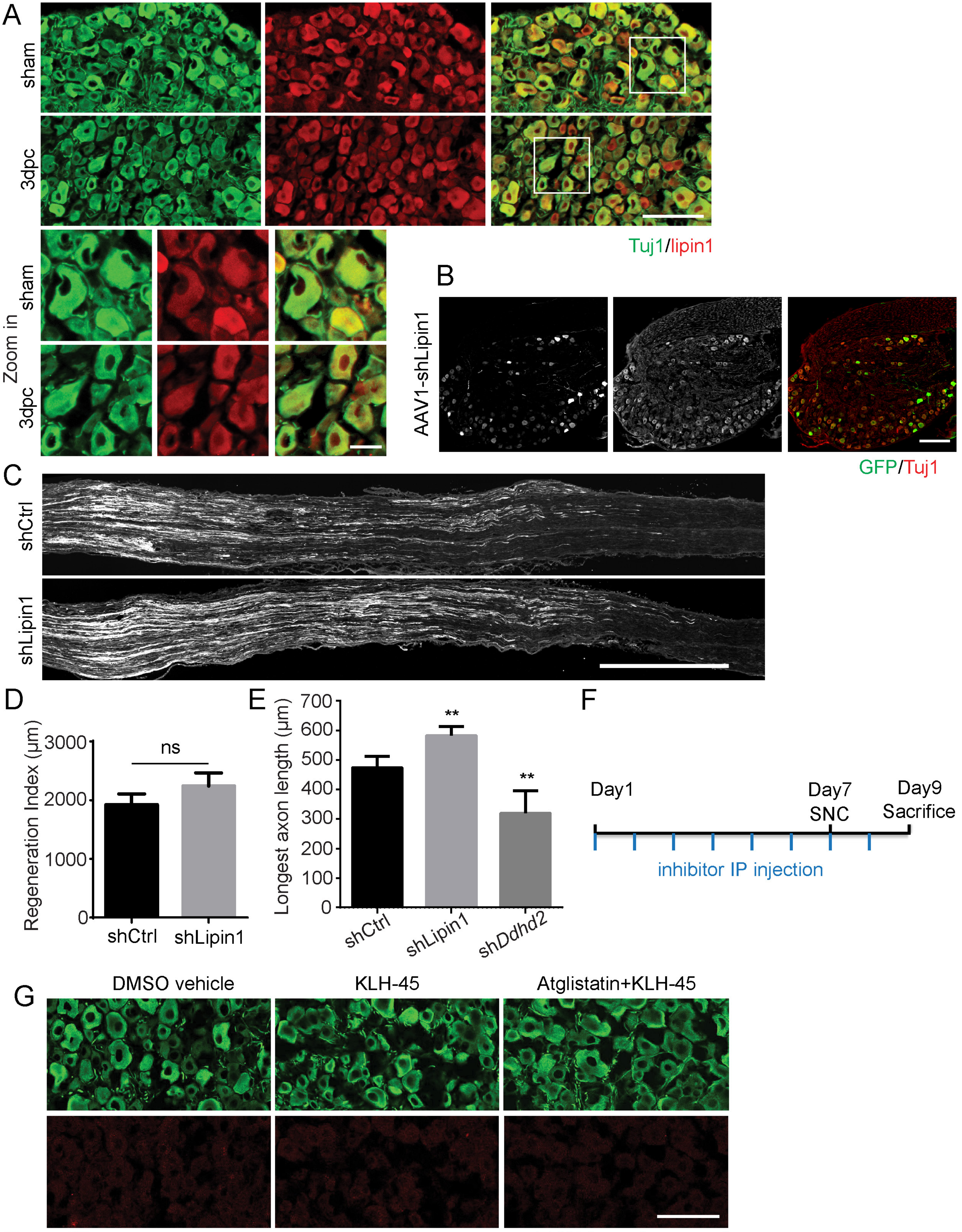
A
sham
3dpc
Tuj1/lipin1
Zoom in sham
Zoom in 3dpc
B
AAV1-shLipin1
GFP/Tuj1
C
shCtrl
shLipin1
D
Regeneration Index (µm)
3000
2000
1000
0
ns
shCtrl
shLipin1
E
Longest axon length (µm)
700
600
500
400
300
200
100
0
**
**
shCtrl
shLipin1
shDdhd2
F
Day1
Day7
SNC
Day9
Sacrifice
inhibitor IP injection
G
DMSO vehicle
KLH-45
Atglistatin+KLH-45
Tuj1/cleaved caspase3

Figure S7

Gene	Knockdown efficiency
Lpin1-shRNA	72.8±13.0%
Dgat1-shRNA	93.7±2.3%
Dgat2-shRNA	97.8±1.3%
Atgl-shRNA	96.4±3.2%
Ddhd2-shRNA	99.2±0.5%
Pcyt1a-shRNA	99.2±0.4%
Pcyt1b-shRNA	60.3±25.5%
Pcyt2-shRNA	85.2±2.6%
Pemt-shRNA	97.1±1.8%
Chka-shRNA	96.8±2.7%
Chkb-shRNA	75.6±6.3%

Table S1: Assessment of shRNA knockdown efficiency, Related to Figure 1, Figure 4, Figure 5 and Figure 6.
Control shRNA and respective shRNAs were transfected to Neuro2A cells. Cells were harvested 72 hrs after transfection and RNA was extracted. Expression levels of corresponding genes were measured by qPCR. Percentages shown are the decrease of corresponding gene expression compared to control shRNA.

Gene	Knockout efficiency
Lpin1-sgRNA	77.9±12.1%
Lpin2-sgRNA	82.3±8.9%
Dgat1-sgRNA	88.5±1.8%
Dgat2-sgRNA	46.5±11.8%

Table S2: Assessment of CRISPR knockout efficiency, Related to Figure 1 and Figure 5.

AAV-sgCtrl or respective sgRNA were injected to Rosa26-Cas9 mice. 4 weeks after injection, retinas from AAV injected mice were harvested and dissociated by papain. Retinal mCherry-positive cells were isolated by FACS and RNA was extracted. We designed primers for qPCR according to our sgRNA sequence to amplify the unchanged wild type mRNA. Percentages shown were calculated from qPCR measurement of unchanged mRNA.

Virus	Expression percentage
AAV2-Lipin1-shRNA	91.2±1.9 % GFP +
AAV2-Lipin1-sgRNA	95.5±1.5 % mCherry +
AAV2-Lipin2-sgRNA	91.6±1.6 % mCherry +
Lipin1-shRNA; Lipin2-sgRNA	86.2±3.6 % mCherry+ GFP+
AAV2-Lipin1-WT	97.7±1.2 % HA-tag +
AAV2-Lipin1-PAPm	97.3±1.3 % HA-tag +
AAV2-Lipin1-ΔNLS	95.2±2.3 % HA-tag +
Lipin1-shRNA; Lipin1-WT	83.9±1.9 % HA-tag + GFP+
Lipin1-shRNA; Lipin1-PAPm	84.1±1.1 % HA-tag + GFP+
Lipin1-shRNA; Lipin1-ΔNLS	81.9±2.3 % HA-tag + GFP+
AAV2-*Atgl*-shRNA	95.5±0.9 % GFP +
Atgl-shRNA; Lipin1-sgRNA	89.7±0.7 % mCherry+ GFP+
AAV2-*Ddhd2*-shRNA	93.1±0.4 % GFP +
Ddhd2-shRNA; Lipin1-sgRNA	86.7±2.7 % mCherry+ GFP+
AAV2-*Dgat1*-sgRNA	94.1±2.3 % mCherry +
Atgl-shRNA; *Dgat1*-sgRNA	83.6±3.9 % mCherry+ GFP+
AAV2-*Dgat2*-sgRNA	93.1±2.6 % mCherry +
Atgl-shRNA; *Dgat2*-sgRNA	84.2±0.3 % mCherry+ GFP+
Lipin1-shRNA; *Dgat1*-sgRNA	85.4±2.3 % mCherry+ GFP+
AAV2-*Chka*-shRNA	91.1±1.1 % GFP +
AAV2-*Chkb*-shRNA	93.1±0.3 % GFP +
AAV2-*Pcyt1a*-shRNA	92.1±1.2 % GFP +
AAV2-*Pcyt1b*-shRNA	88.9±0.9 % GFP +
AAV2-*Pcyt2*-shRNA	89.7±1.2 % GFP +
AAV2-*Pemt*-shRNA	90.9±2.5 % GFP +
Chka-shRNA; Lipin1-sgRNA	86.4±3.2 % mCherry+ GFP+
Chkb-shRNA; Lipin1-sgRNA	85.3±1.8 % mCherry+ GFP+
Pcyt1a-shRNA; Lipin1-sgRNA	84.8±2.2 % mCherry+ GFP
Pcyt1b-shRNA; Lipin1-sgRNA	84.7±1.6 % mCherry+ GFP+
Pcyt2-shRNA; Lipin1-sgRNA	83.7±1.1 % mCherry+ GFP+
Pemt-shRNA; Lipin1-sgRNA	82.8±3.2 % mCherry+ GFP+
AAV2-*Pcyt1a*-WT	93.4±1.3 % HA-tag +
AAV2-*Pcyt1a*-CA	91.3±2.2 % HA-tag +
AAV2-*Pcyt1b*	94.8±1.0 % HA-tag +
AAV2-*Pcyt2*	92.5±2.5 % HA-tag +

Table S3. Assessment of virus expression efficiency for in vivo experiment, Related to Figure 1, Figure 3, Figure 4, Figure 5 and Figure 6. Respective viruses were injected to wildtype mice. 4 weeks after injection, retinas were harvested and then immunostained by HA-tag and Tuj1 antibody. Percentages shown were calculated by the ratio of GFP, mCherry or HA-tag positive cells to Tuj1 positive cells.

Sequence for shRNA	
Control shRNA	GACCATCAATATGACTAGA
Lipin1 shRNA:	CGGAACTCTGTAGACAGAAT
Dgat1 shRNA	GCCCTTCAAGGATATGGACT
Dgat2 shRNA:	GCTACTTCCGAGACTACTTT
Atgl shRNA	GTGAAGCAGGTGCCAACATTA
Ddhd2 shRNA	GAAAGAAGATACTGAACCA
Chka shRNA	GAGGCCGACTGGAGCAGTTTAT
Chkb shRNA	GTGAGTGGGTTTATGATTATA
Pemt shRNA	GTTGACGGTGCTGGTGGCAA
Pcyt1a shRNA	GTCACTGTGATGAACGAGAATG
Pcyt1b shRNA	GCCAGGTACAAACAGACACTTA
Pcyt2 shRNA	CTGCTATGACATGGTGCATTA
**Sequence for sgRNA**	
Control Lacz sgRNA	GCGTCGTGACTGGGAAAACCC
Lipin1 sgRNA1	GGTTCAGACAATGAATTACG
Lipin1 sgRNA2	GTTCAGACAATGAATTACGT
Lipin2 sgRNA1	GGTTATATATCCGGATCACGAGG
Lipin2 sgRNA2	GTACGTGAAAAGGCGAGCACTGG
Dgat1 sgRNA1	CTCAACTACGATGCCCCAG
Dgat1 sgRNA2	GATCTTGCAGACGATGGCACC
Dgat2 sgRNA1	GATTTGGCCTTCCAGAGACTG
Dgat2 sgRNA2	GCCCGGAGTAGGCGGCGATGA

Table S4. Sequence information of shRNA and sgRNA, Related to Figure 1, Figure 4, Figure 5 and Figure 6.

**Sequence for qPCR primers**	
Lipin1 qPCR primer	Foward: ATGAATTACGTGGGGCAGC<br><br>Reverse: CCACTTTCTCTCGGGAGCGG
Lipin2 qPCR primer	Foward: AACCGTTACTACAACTGGGC<br><br>Reverse: CGCCAAAACCACCATCGACC
Dgat1 qPCR primer	Foward: GGTGCCATCGTCTGCAAGAT<br><br>Reverse: ACCAGGATGCCATACTTGAT
Dgat2 qPCR primer	Foward: ACCCACCCCTTCTAGCGTTC<br><br>Reverse: TCTCAAGAATCCCTGGAGTCAC
Atgl qPCR primer	Foward: GACAGCTCCACCAACATCCA<br><br>Reverse: CCTTCGAGAGGCGGTAGAGA
Ddhd2 qPCR primer	Foward: GCCAGGAAGAATTCATTTGACCC<br><br>Reverse: TGGTGTGGGATCAGCATTGG
Pcyt1a qPCR primer	Foward: CCGTAAACCAACTGCGCAAC<br><br>Reverse: GGGACTCTGACCTCGGAGAG
Pcyt1b qPCR primer	Foward: GTCAGGGTCTTGCGTGAGAA<br><br>Reverse: CTGGGCATATCAAGCTGCCT

Pcyt2 qPCR primer	Foward: ATCTCCTGGCTGCTGTGATG<br><br>Reverse: AGGCTGGGAGGTACAGAGAG
Pemt qPCR primer	Foward: CTCCCATCTCGCTACCACAT<br><br>Reverse: CTGTGGAGGCTTCGGCAATA
Chka qPCR primer	Foward: GTTCTCCTGGCCTTCCAACA<br><br>Reverse: CGAGAACCTGAGGTCATTGCT
Chkb qPCR primer	Foward: GGAGGCTCCAGGAGAAACTTGA<br><br>Reverse: ATCAGTCGGTACTCCCTGGCA
Gapdh qPCR primer	Foward: GGAGAGTGTTTCCTCGTCCC<br><br>Reverse: ATGAAGGGGTCGTTGATGGC
**Sequence for PCR primers in T7E1 assay**	
Lipin1 PCR primer for T7E1 assay	Foward: CTGGGGTGCTGCTTGTGTTA<br><br>Reverse: GGGCTTCTGAAACTAGGCCAT
Dgat1 PCR primer for T7E1 assay	Foward: CCTGACTGCAGTTGGTTTCT<br><br>Reverse: CCTGTAAAACAGTGGCTGGT
Dgat2 PCR primer for T7E1 assay	Foward: TTTCCCATGCTGCCCGTAGC<br><br>Reverse: TGGTACGAGGAACCCGACC

Table S5. Sequence information of PCR primers, Related to Figure 1, Figure 4, Figure 5 and Figure 6.
